# miR-29a-3p and TGF-β Axis in Fanconi anemia: mechanisms driving metabolic dysfunction and genome stability

**DOI:** 10.1007/s00018-025-05775-w

**Published:** 2025-06-25

**Authors:** Nadia Bertola, Stefano Regis, Vanessa Cossu, Matilde Balbi, Martina Serra, Fabio Corsolini, Cristina Bottino, Paolo Degan, Carlo Dufour, Filomena Pierri, Enrico Cappelli, Silvia Ravera

**Affiliations:** 1 IRCCS Ospedale Policlinico San Martino, Largo Rosanna Benzi, 10, Genova, 16132 Italy; 2 Laboratory of Clinical and Experimental Immunology, IRCCS Istituto Giannina Gaslini, Via Gerolamo Gaslini 5, Genoa, 16147 Italy; 3 Experimental Medicine Department, University of Genova, Via De Toni 14, Genoa, 16132 Italy; 4 Haematology Unit, IRCCS Istituto Giannina Gaslini, Via Gerolamo Gaslini 5, Genoa, 16147 Italy

**Keywords:** DNA repair, Fanconi anemia, Inflammation, MiR-29a-3p, Mitochondrial metabolism

## Abstract

Fanconi anemia (FA) is a genetic disorder characterized by bone marrow failure and cancer predisposition. The FA cellular phenotype is marked by a defective DNA double-strand break repair. Alongside this defect, FA cells exhibit mitochondrial dysfunction and redox unbalance. In addition, FA cells display an altered microRNA profile, including miR-29a-3p, which plays a crucial role in hematopoiesis by supporting the self-renewal, lineage commitment, and differentiation of hematopoietic stem cells (HSCs). In this study, we demonstrate that miR-29a-3p is downregulated in lymphoblasts and fibroblasts mutated for the FANC-A gene, leading to hyperactivation of PI3K/AKT pathway due to the overexpression of its target genes, FOXO3, SGK1, and IGF1, and resulting in altered mitochondrial metabolism and insufficient antioxidant response. In addition, miR-29a-3p downregulation appears associated with hyperactivation of the TGF-β signal. By contrast, FA cells transfected with miR-29a-3p show an improvement in mitochondrial metabolism, oxidative stress response, and DNA damage accumulation, by inhibiting the PI3K/AKT pathway and modulating the TGF-β pathway through a feedback mechanism. In conclusion, our results highlight the central role of miR-29a-3p in FA cells, suggesting that it is a promising molecular target to address several mechanisms based on FA pathogenesis.

**Graphical abstract:**

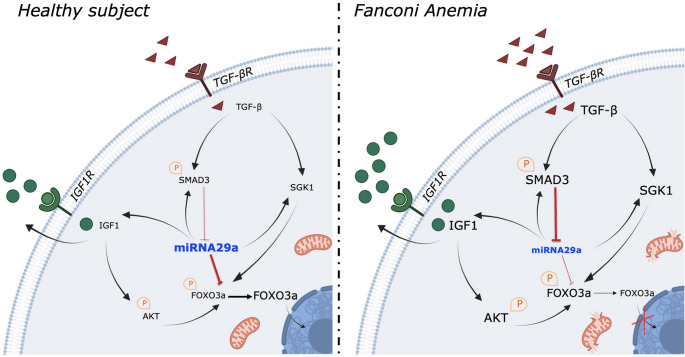

**Supplementary Information:**

The online version contains supplementary material available at 10.1007/s00018-025-05775-w.

## Introduction

Fanconi anemia (FA) is a genetic disease, typically with pediatric onset, characterized by aplastic anemia and predisposition to several types of cancer, such as acute myeloid leukemia, gynecological carcinoma, and head and neck squamous cell carcinoma (HNSCC) [1, 2].

DNA repair has long been considered the primary defect in FA cells [3, 4]. However, in the last 20 years, research has shown that FA proteins are involved in several other cellular processes, including defective energy metabolism [5, 6], impaired antioxidant response [7–9], and the overproduction of pro-inflammatory and cytotoxic cytokines [10–13]. In detail, FA cells are characterized by altered electron transport between respiratory complexes I and III [14, 15], leading to the uncoupling of oxidative phosphorylation (OxPhos) [16–18]. As a result, FA cells exhibit increased reactive oxygen species (ROS) production [19, 20], not counteracted by endogenous antioxidant defensesbecause FA cells are not able to trigger an adaptative response to the oxidative stress increment [21–23]. This imbalance in redox status promotes DNA damage accumulation [24] and the development of a pro-inflammatory environment [7], triggering a vicious circle. Additionally, unrepaired DNA damage is reported to contribute to the pro-inflammatory conditions observed in FA patients [25, 26], increasing the expression of cytotoxic cytokines. This heightened expression is associated with increased cellular sensitivity and leads to the death of hematopoietic progenitors [13, 27]. In this context, several pro-inflammatory cytokines have already been associated with defective hematopoiesis in patients with FA [28]. For example, bone marrow mononuclear cells (BM-MNC) isolated from FA patients have shown hypersensitivity to TNF-α, which inhibits erythropoiesis in vitro [12]. Additionally, hyperactivity of the TGF-β/SMAD3 pathway inhibits DNA repair via homologous recombination (HR), activating non-homologous end joining (NHEJ), which is toxic to FA HSCs [29]. Although DNA damage accumulation, impaired energy metabolism and antioxidant defenses, and increased production of proinflammatory cytokines in FA have been extensively described in the literature, no mechanism has yet been proposed to explain their interconnection and mutual influence. To address this gap, we focused our attention on the microRNA (miRNA) profile in FA cells, because, to date, few studies have explored the miRNA profile in FA cells [30–32], although their modulation appears to play a role in FA. For example, an initial analysis of the miRNA profile in FA by the Cappelli and Degan group reported a total of 24 miRNAs that were commonly altered in both FA cell lines and patient samples compared to healthy subjects, which were involved in seven biological pathways associated with the FA phenotype, such as DNA damage protection, gene stability, and fatty acid metabolism [30]. A second analysis also revealed the altered expression of miR-1246 and miR-206, two miRNAs that influence various biochemical pathways associated with processes, such as autophagy, oxidative stress response, and cell cycle regulation. In particular, miR-206 was identified as a key player in the BM mesenchymal stem cells (MSC) regulation in FA patients [31]. Additionally, Cagnan et al. identified 50 microRNAs with differential expression between healthy donors and FA patients prior to bone marrow transplantation (BMT), five of which (e.g., hsa-miR-146a-5p, hsa-miR-148b-3p, hsa-miR-187-3p, hsa-miR-196b-5p, and hsa-miR-25-3p), involved in senescence and cancer progression, were significantly downregulated in FA patients. Interestingly, after BMT, the expression of hsa-miR-146a-5p and hsa-miR-874-3p was restored, suggesting a potential role in the recovery of bone marrow function [32].

In this study, we investigate the role of miR-29a-3p, a member of the miR-29 family. The miR-29 family members plays a multifaceted role in several cellular processes and diseases [33], primarily acting as tumor suppressors [33, 34], anti-fibrotic agents bytheTGF-β signal modulation [35, 36], and regulators of immune responses [37, 38]. miR-29s are involved in processes such as apoptosis, proliferation, and differentiation. For example, miR-29s can act as tumor suppressors by targeting oncogenes and anti-apoptotic genes, ultimately inhibiting tumorigenesis and metastasis [39]. Additionally, miR-29s can influence epigenetic mechanisms [40], such as DNA methylation and histone modifications, affecting gene expression; it have been linked to impaired glucose metabolism [41]. More sepcifically, miR-29a-3p modulates numerous biochemical and physiological pathways, including the maturation, differentiation, and survival of hematopoietic stem and progenitor cells (HSPCs) [42]. In detail, miR-29a-3p has been shown to regulate DNA methyltransferase 3 (DNMT3), supporting self-renewal in HSPCs and guiding lineage commitment and differentiation [42–44]. Interestingly, the expression of miR-29a-3p is inhibited by the hyperactivation of the TGF-β pathway [45], whichis a hallmark of FA cells [46]. In addition, altered miR-29a-3p expression is associated with the development of Head and Neck Squamous Cell Carcinoma (HNSCC) [47], a type of cancer highly prevalent in individuals with FA [48].

Our findings reveal that FA lymphoblasts and primary fibroblasts exhibit significantly reduced expression of miR-29a-3p compared to healthy controls and isogenic corrected cells. This reduction is mediated by negative feedback involving TGF-β pathway activity. Furthermore, transfection of FA cells with miR-29a-3p restores mitochondrial function, enhances the oxidative stress response and reduces DNA damage by modulating the TGF-β pathway through decreasing SMAD3 phosphorylation.

## Materials and methods

### Samples

Fanc-A lymphoblast cell lines and FANC-A primary fibroblast cell lines that carried out different mutations of the FANC-A gene were obtained from the ‘‘Cell Line and DNA Biobank from Patients affected by Genetic Diseases’’ (G. Gaslini Institute) - Telethon Genetic Biobank Network (Project No. GTB07001). As controls, isogenic FA-corr cell lines generated by the same FANC-A lymphoblast and fibroblast cell lines corrected with S11FAIN retrovirus (Lympho FA-corr and Fibro FA-corr) were employed [49]. None of the samples used in this study were affected by HNSCC or pre-malignant changes.

The study was conducted following the Declaration of Helsinki and approved by the regional ethics committee, protocol JS002, register number 037 − 21/01/2019. All the subjects or their legal guardians gave written informed consent to the investigation.

### Cell culture and treatments

For lymphoblast cell lines, RPMI-1640 medium (GIBCO, Billing, MT, USA) containing 10% fetal bovine serum (FBS, Euroclone, Milano, Italy), 100 U/mL penicillin, and 100 µg/mL streptomycin (Euroclone, Milano, Italy) was used, and the cells were grown at 37 °C with a 5% CO_2_ [49]. Primary fibroblasts were cultured as a monolayer in DMEM high glucose with glutamax^®^ (GIBCO, Billing, MT, USA), containing 10% FBS (Euroclone, Milano, Italy), 100 U/mL penicillin, and 100 µg/mL streptomycin (Euroclone, Milano, Italy) at 37 °C with a 5% CO_2_ [49].

### FA lymphoblast and fibroblast cells transfection with miR-29a-3p

FA lymphoblast cells were transfected with miR-29a-3p mimic (Thermo Fisher Scientific, Waltham, MA, USA), using the lipofectamine RNAiMAX Transfection Reagent (Invitrogen, Waltham, MA, USA) according to the manufacturer’s instruction. In detail, for subsequent RNA extraction, 500.000 cells grown in 6-well plates were transfected with 25 pmol of miR-29a-3p mimic or negative control mimic using 7.5 µl lipofectamine. After 48 h, cells were harvested, washed with PBS, lysed, and RNA was extracted. For the subsequent biochemical analysis, 7.5 × 10^6^ cells grown in 75 cm^2^ flasks were transfected with 187.5 pmol of miR-29a-3p mimic or negative control mimic using 56.25 µl lipofectamine. Cells were processed 48 h post-transfection.

FA fibroblast cells were transfected with miR-29a-3p mimic (Thermo Fisher Scientific, Waltham, MA, USA) using the same transfection reagent (Invitrogen, Waltham, MA, USA). In detail, for the subsequent RNA extraction, 75,000 cells grown in 6-well plates were transfected with 25 pmol of miR-29a-3p mimic or negative control mimic using 7.5 µl lipofectamine. After 48 h, cells were harvested, washed with PBS, lysed, and RNA was extracted. For subsequent biochemical analysis, 500,000 cells grown in 175 cm^2^ flasks were transfected with 437.5 pmol of miR-29a-3p mimic or negative control mimic using 131.25 µl lipofectamine. Cells were processed 48 h post-transfection.

### FA lymphoblast cell treatments

To inhibit the TGF-β pathway, lymphoblasts were treated with Luspatercept, an inhibitor acting on the SMAD2/3 signaling [50]. In detail, 500,000 cells were plated in a 2 ml culture medium in the presence of 10 µg/ml Luspatercept for 48 h.

To inhibit IGF1 signaling, 500,000 lymphoblasts were treated with 400 pM Klotho [51] for 48 h.

### In silico selection of pathway-related miR-29a-3p target genes

To identify putative miR-29a-3p target genes potentially involved in Fanconi cell metabolism impairment, we utilized the miRPathDB v2.0 database (https://mpd.bioinf.uni-sb.de). A curated list of miR-29a-3p-regulated genes associated with DNA damage response, oxidative stress, mitochondrial metabolism, lipid metabolism, and apoptosis was generated by evaluating the strength of predicted miRNA-target gene interactions using TargetScan (https://mpd.bioinf.uni-sb.de). Further refinement was conducted by assessing the role and the subcellular localization of each gene with the help of the NCBI Gene database (https://www.ncbi.nlm.nih.gov/gene) and GeneCards (https://www.genecards.org).

### RNA isolation to evaluate the expression of miR-29a-3p and FOXO3, SGK1, and IGF1 genes

RNA, including the small RNA fraction, was extracted using the RNeasy Plus Mini Kit (Qiagen, Hilden, Germany) according to the manufacturer’s protocol. The expression of miR-29a-3p was assessed using a specific TaqMan MicroRNA Assay (Applied Biosystems, Waltham, MA, USA). Briefly, 10 ng of RNA was reverse-transcribed using the TaqMan MicroRNA Reverse Transcription Kit and the specific RT primer. Real-time PCR was performed in triplicate with specific primers. miRNA expression levels were normalized to RNU44 expression.

To evaluate the expression of the FOXO3, SGK1, and IGF1 genes, 100 ng of RNA were reverse transcribed using the SuperScript VILO IV cDNA Synthesis Kit (Invitrogen, Waltham, MA, USA). The resulting cDNA was used for real-time PCR with primers provided in the specific TaqMan Gene Expression Assays (Applied Biosystems, Waltham, MA, USA). Gene expression levels were normalized to GAPDH expression. All experiments were performed in triplicate. Ct standard deviation values of used reference genes among the performed treatments are reported in Supplementary Table 1.

### Catalase activity evaluation

Catalase (CAT) activity was assayed as a marker of cellular antioxidant defenses. For each spectrophotometric assay, 50 µg of total proteins were used, following the H_2_O_2_ decomposition at 240 nm. The assay mix contained 100 mM Tris-HCl (pH 7.4, Sigma-Aldrich, St. Louis, MO, USA) and 5 mM H_2_O_2_ (Sigma-Aldrich, St. Louis, MO, USA) [21].

### Oxidative stress markers evaluation

The thiobarbituric acid reactive substances (TBARS) assay was employed to quantify malondialdehyde (MDA), an indicator of lipid peroxidation. The TBARS reagent was prepared using 0.25 M HCl, 0.25 mM trichloroacetic acid, and 26 mM thiobarbituric acid (all sourced from Merck, Darmstadt, Germany). A total of 50 µg of protein, dissolved in 300 µl of Milli-Q water, was mixed with 600 µl of the TBARS solution. The reaction mixture was incubated at 95 °C for 1 h and the absorbance was measured spectrophotometrically at 532 nm. Standard solutions of MDA, with concentrations ranging from 1 to 20 µM, were used to generate a calibration curve [21].

To measure 8-hydroxy-2-deoxyguanosine (8-OHdG), a biomarker of oxidative DNA damage, an ELISA kit (#ab201734, Abcam, Cambridge, UK) was utilized according to the manufacturer’s instructions.

### Evaluation of aerobic metabolism function and efficiency

The OxPhos function was assessed by measuring the oxygen consumption rate (OCR) and F_o_F_1_-ATP synthase activity.

OCR was determined using an amperometric electrode (Unisense Microrespiration, Aarhus, Denmark) in a closed chamber. For each test, 10^5^ cells were permeabilized for 1 min with 0.03 mg/ml digitonin and then used in the assay. To activate the respiratory pathway led by Complex I or Complex II, 10 mM pyruvate with 5 mM malate (Merck, Darmstadt, Germany) or 20 mM succinate Merck, Darmstadt, Germany) were added, respectively [21].

F_o_F_1_-ATP synthase activity was measured in 10^5^ cells suspended in PBS plus 0.6 mM ouabain Merck, Darmstadt, Germany) and 0.25 mM di (adenosine)−5-penta-phosphate (an adenylate kinase inhibitor, Merck, Darmstadt, Germany). After 10 min of incubation, 10 mM pyruvate with 5 mM malate (Merck, Darmstadt, Germany) or 20 mM succinate (Merck, Darmstadt, Germany) were added to stimulate pathways mediated by Complex I or II, respectively. ATP production was quantified using a luminometer (GloMax^®^ 20/20 Luminometer, Promega Italia, Milan, Italy), employing the luciferin/luciferase chemiluminescent method (ATP bioluminescence assay kit CLS II, #11699695001 Roche, Basel, Switzerland). Measurements were taken at 30-second intervals over 2 min [21].

The OxPhos efficiency was determined by calculating the P/O ratio, which represents the amount of ATP synthesized aerobically per oxygen molecule consumed. Mitochondria with optimal efficiency exhibit P/O ratios around 2.5 for pyruvate and malate or 1.5 for succinate. Ratios below these thresholds suggest incomplete oxygen utilization for ATP production, potentially reflecting increased ROS generation [21].

### Assessment of ATP and AMP intracellular concentration and the consequent cellular energy status

ATP and AMP concentrations were assayed in 50 µg of total protein. ATP content spectrophotometric analysis was performed following the NADPreduction at 340 nm. The assay solution contained 100 mMTris-HCl (pH 8.0; Merck, Darmstadt, Germany), 0.2 mM NADP (Merck, Darmstadt, Germany), 5 mM MgCl_2_ (Merck, Darmstadt, Germany), 50 mM glucose (Merck, Darmstadt, Germany), and 3 µg of pure hexokinase and glucose-6-phosphate dehydrogenase (Merck, Darmstadt, Germany) [20].

AMP was measured spectrophotometrically following the NADH oxidation at 340 nm. The reaction medium was composed of 100 mM Tris-HCl (pH 8.0; Merck, Darmstadt, Germany), 5 mM MgCl_2_ (Merck, Darmstadt, Germany), 0.2 mM ATP (Merck, Darmstadt, Germany), 10 mMphosphoenolpyruvate (Merck, Darmstadt, Germany), 0.15 mM NADH (Merck, Darmstadt, Germany), 10 IU adenylate kinase, 25 IU pyruvate kinase, and 15 IU lactate dehydrogenase (Merck, Darmstadt, Germany) [20].

The cellular energy status was calculated as the ratio between intracellular concentration of ATP and AMP (ATP/AMP ratio) [20].

### Evaluation of electron transfer between complex I and complex III

To evaluate the electron transfer between Complex I and Complex III, a spectrophotometric assay was performed following the reduction of oxidized cytochrome *c* (cyt*c*) at 550 nm in the presence of NADH. The assay medium contained 50 mMTris-HCl (pH 7.4; Merck, Darmstadt, Germany), 5 mMKCl (Merck, Darmstadt, Germany), 2 mM MgCl_2_ (Merck, Darmstadt, Germany), 0.5 M NaCl (Merck, Darmstadt, Germany), 0.03% oxidized cyt*c* (Merck, Darmstadt, Germany), and 0.6 mM NADH (Merck, Darmstadt, Germany) [20].

### Western blot analysis

The expression of several proteins belonging to the FOXO3a, SGK1, IGF1, and SMAD3 (TGF-β effector) signalling pathways was assessed by western blot (WB) analysis performed on cell homogenates of FA lymphoblasts and their controls. In detail, denaturing electrophoresis (SDS-PAGE) was performed on 30 µg of proteins employing a 4–20% gradient gel (BioRad, Hercules, CA, USA). The following primary antibodies were used: phospho-H2AX (#05–636, Merck, Darmstadt, Germany). phospho-FOXO3a (#9466,Cell Signaling Technology, Beverly, MA, USA), FOXO3a (#2497S, Cell Signaling Technology, Beverly, MA, USA), GAPDH (#2118, Cell Signaling Technology, Beverly, MA, USA), Histone H3 (#4499, Cell Signaling Technology, Beverly, MA, USA), phospho-AKT (#4060S, Cell Signaling Technology, Beverly, MA, USA), AKT (#4691S, Cell Signaling Technology, Beverly, MA, USA), phospho-SGK1 (#5599, Cell Signaling Technology, Beverly, MA, USA), SGK1 (#711183, Thermo Fisher Scientific, Waltham, MA, USA), phospho-SMAD3 (#9520S, Cell Signaling Technology, Beverly, MA, USA), SMAD3 (#9523S, Cell Signaling Technology, Beverly, MA, USA), and β-Actin (#MA1-140, ThermoFisher Scientific, Waltham. MA, USA). All primary antibodies were diluted following the manufacturer’s instructions in PBS plus 0.15% Tween 20 (PBSt; Roche, Basel, Switzerland). Specific secondary antibodies were employed (Merck, Darmstadt, Germany, all diluted 1:10,000 in PBSt). Bands were detected in the presence of an enhanced chemiluminescence substrate (ECL, BioRad, Hercules, CA, USA), by a chemiluminescence system (Alliance 6.7 WL 20 M, UVITEC, Cambridge, UK). Band intensity was evaluated by UV1D software (UVITEC, Cambridge, UK). All bands of interest were normalized versus the actin signal detected on the same membrane.

Since FOXO3a function is closely linked to its translocation from the cytoplasm to the nucleus [52], FOXO3a expression has also beeen evalauted in nuclear fraction obtained from FA and FAcorr lymphoblatsts. To obtain nuclei-enriched fractions (N), cells were homogenated in a 0.25 M sucrose solution. This homogenate (H) was centrifuged at 800 g for 10 min. The supernatant was collected and used as a cytoplasmic fraction (C), and the pellet was resuspended in 0.25 M sucrose solution and centrifuged again at 800 g for 10 min to obtain N.

In addition, the expression of proteins involved in the TGF-β signalling modulation has been evaluated in samples treated with Luspatercept, a TGF-β pathway inhibitor acting on SMAD2/3 signaling [50].

### Statistical analysis

Data about gene expression, western blot analysis, ATP synthesis, OCR, mitochondrial efficiency were obtained from three independent replicate experiments; the other biochemical data were obtained from six independent replicate experiments. Data were shown as means ± standard deviation (SD). Student’s t test or one-way ANOVA followed by Tukey’s multiple comparison test by Prism 9 Software (GraphPad Software Inc., Boston, MA, USA) was used to compare the differences between two or more groups. *p* < 0.05 was considered statistically significant. ns represent statistically non-significant, * *p* < 0.05, ** *p* < 0.01, *** *p* < 0.001, and **** *p* < 0.0001.

## Results

### miR-29a-3p expression is downregulated in Fanc-A cells

**Fig. 1:**
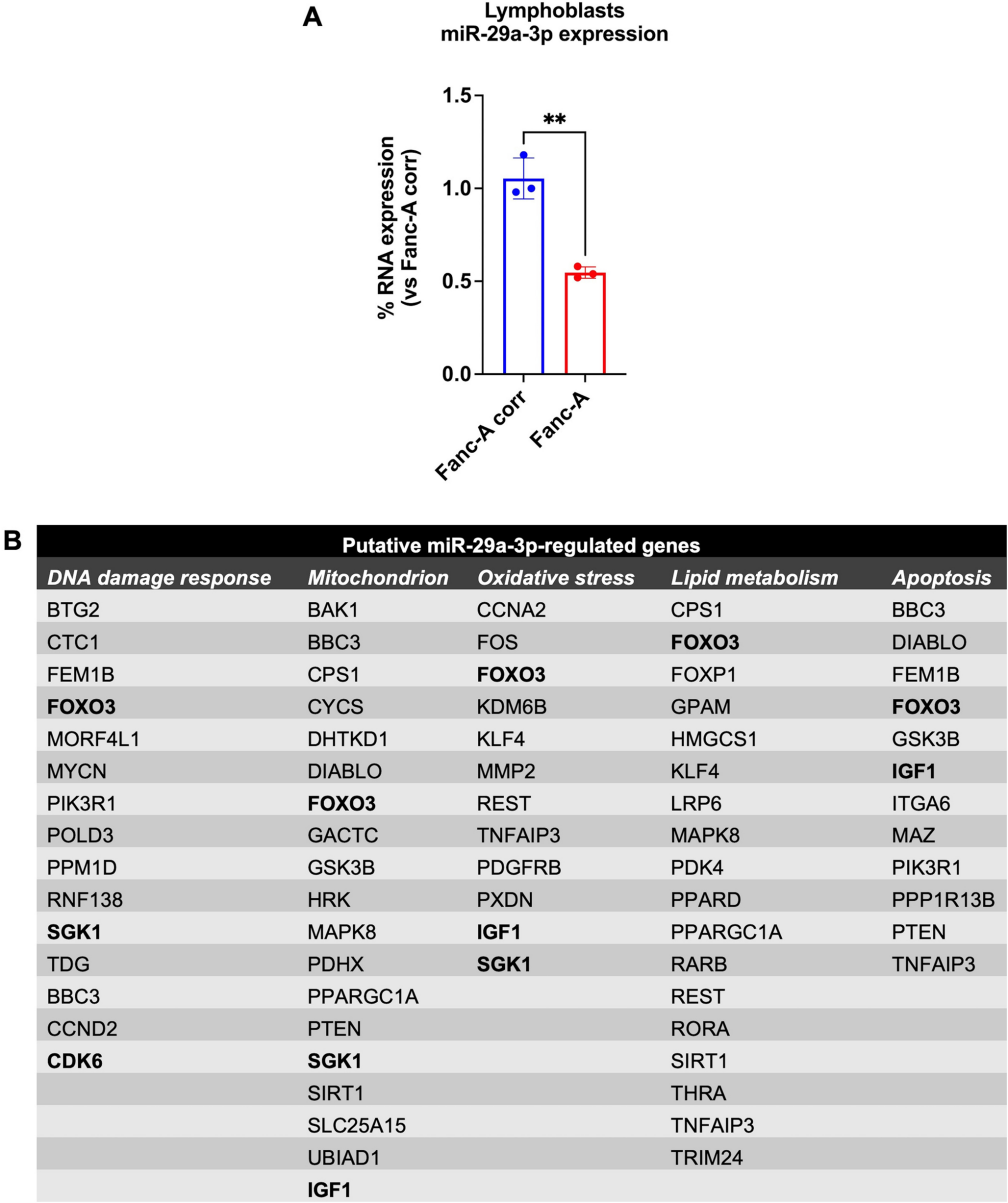
miR-29a-3p expression in Fanc-A cells and putative miR-29a-3p-regulated genes. (**A**) The graph shows the comparison of miR-29a-3p expression in isogenic Fanc-A corrected lymphoblasts (Fanc-A corr, used as a control) and Fanc-A lymphoblasts (Fanc-A). RNU44 was used as a reference control. Data are expressed as mean ± SD and are representative of three independent experiments (*n* = 3). (**B**) Putative target genes of miR-29a-3p in DNA damage response processes, mitochondrial function, oxidative stress, lipid metabolism, and apoptosis.In bold are marked the genes that are recurrent across the different pathways analyzed. In panel A, ** indicates a significant difference for *p* < 0.01

Although several miRNAs may potentially be involved in the pathogenesis of FA [30, 31], we focused our attention on miR-29a-3p, due to its role in modulating mitochondrial activity and redox balance [42], two features altered in FA cells [17, 21]. Our data show a significant reduction in miR-29a-3p expression in Fanc-A lymphoblasts (Fig. 1A) and fibroblasts (Figure S1) compared to the corresponding isogenic corrected cells, suggesting a possible role of miR-29a-3p in mitochondrial dysfunction and associated oxidative stress production in FA cells.

### Several putative miR-29a-3p-regulated genes are involved in DNA repair, mitochondrial function, redox balance, and apoptosis

Based on the results of miR-29a-3p expression, we interrogated the miRPathDB v2.0 database to identify putative associations between miRNAs, target genes, and cellular pathways. Specifically, we selected genes potentially regulated by miR-29a-3p and involved in DNA damage response, oxidative stress, mitochondrial metabolism, lipid metabolism, and apoptosis, all of which are pathways potentially altered in FA cells. Furthermore, TargetScan was used to define the gene list based on the strength of predicted miRNA-target gene interactions. The list was then refined by analyzing the role and subcellular location of each gene using NCBI Gene and GeneCards. The final list is reported in Fig. 1B.

### miR-29a-3p transfection reduced the oxidative damage and improved the oxidative phosphorylation in Fanc-A cells

Since low expression of miR-29a-3p in FA cells suggests possible altered expression of genes involved in oxidative stress response (Fig. 1B), catalase (CAT) activity, and intracellular malondialdehyde (MDA) levels have been evaluated as markers of antioxidant defenses and oxidative damage, in FA cells transfected with miR-29a-3p. Additionally, 8-hydroxy-2’-deoxyguanosine (8-OHdG) content and histone H2AX phosphorylation have been assayed as DNA damage markers.

**Fig. 2:**
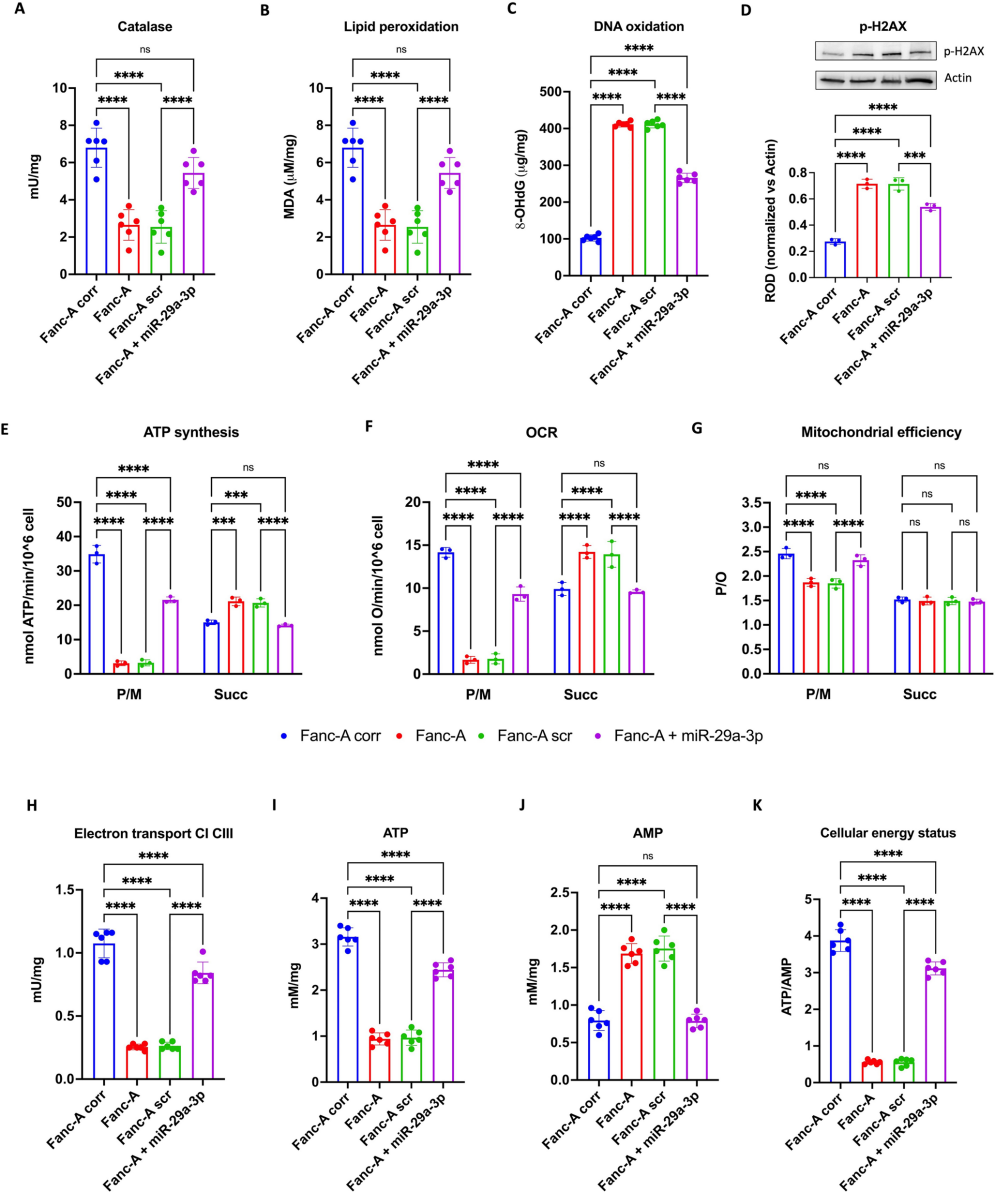
Antioxidant defenses, oxidative stress, and energy metabolism were modulated by miR-29a-3p expression in Fanc-A lymphoblasts. All analyses were conducted on Fanc-A lymphoblasts corrected with the WT Fanc-A gene (Fanc-A corr), Fanc-A lymphoblasts (Fanc-A), Fanc-A lymphoblasts transfected with a miRNA mimic negative control for 48 h (Fanc-A scr), and Fanc-A lymphoblasts transfected with miR-29a-3p for 48 h (Fanc-A + miR-29a-3p) (**A**) Catalase activity as an antioxidant defense marker. (**B**) Intracellular concentration of malondialdehyde (MDA) as a lipid peroxidation marker. (**C**) 8-hydroxy-2’-deoxyguanosine (8-OHdG) content as a DNA oxidation marker. (**D**) WB signal and relative densitometric analysis of p-H2AX. The densitometric analysis was normalized to the actin signal and used as a housekeeping protein. (**E**) ATP synthesis through F_o_F_1_-ATP synthase. (**F**) Oxygen consumption rate (OCR). (**G**) P/O value, an OxPhos efficiency marker. For Panels E, F, and G, the analyses were conducted in the presence of pyruvate plus malate (P/M) or succinate (Succ) to induce the OxPhos pathways led by Complex I or Complex II, respectively. (**H**) Electron transfer between Complexes I and III. (**I**) Intracellular ATP content. (**J**) Intracellular AMP content. (**K**) Cellular energy status is obtained by calculating the ATP/AMP ratio Data are expressed as mean ± SD and are representative of three independent experiments (*n* = 3) for Panels D-G and six independent experiments for Panels A-C and H-K (*n* = 6).***, and **** indicate a significant difference for *p* < 0.001, and 0.0001, respectively. ns indicates a no-significant statistical difference

Fanc-A cells displayed reduced CAT activity (Fig. 2A and Figure S2A) and increased concentrations of MDA (Fig. 2B and Figure S2B) and 8-OHdG (Fig. 2C and Figure S2C) compared to the corrected cells, indicating increased oxidative damage to lipids and DNA due to defective antioxidant defenses. In addition, histone H2AX was hyper-phosphorylated in Fanc-A lymphoblasts (Fig. 2D), confirming DNA damage accumulation. However, these detrimental effects appeared partially recovered after miR-29a-3p transfection, suggesting a role of this miRNA in the unbalanced oxidative stress of FA cells.

Considering that miR-29a-3p could also regulate genes involved in energy metabolism and that FA cells display an altered OxPhos associated with increased oxidative stress production [14, 17, 49], the ATP synthesis through F_o_F_1_-ATP synthase, the oxygen consumption rate (OCR), and the OxPhos efficiency were investigated before and after miR-29a-3p transfection in Fanc-A lymphoblasts and fibroblasts. As expected, data show that Fanc-A cells were characterized by defective ATP production (Fig. 2E and Figure S2D) and OCR (Fig. 2F and Figure S2E) when OxPhos was induced by pyruvate/malate addition but not with succinate. This impairment resulted in a decrease of OxPhos efficiency via complexes I-III-IV, as indicated by the reduction of the P/O value (Fig. 2G and Figure S2F). All these metabolic parameters showed a significant improvement after miR-29a-3p transfection. This recovery depended on the restoration of electron transport between respiratory complexes I and III (Fig. 2H and Figure S2G), leading to an increase in intracellular ATP content (Fig. 2I and Figure S2H) and a reduction in AMP concentration (Fig. 2J and Figure S2I), ultimately improving the energy status (Fig. 2K and Figure S2J).

### miR-29a-3p transfection restores FOXO3, SGK1, and IGF1 genes expression in Fanc-A cells

**Fig. 3:**
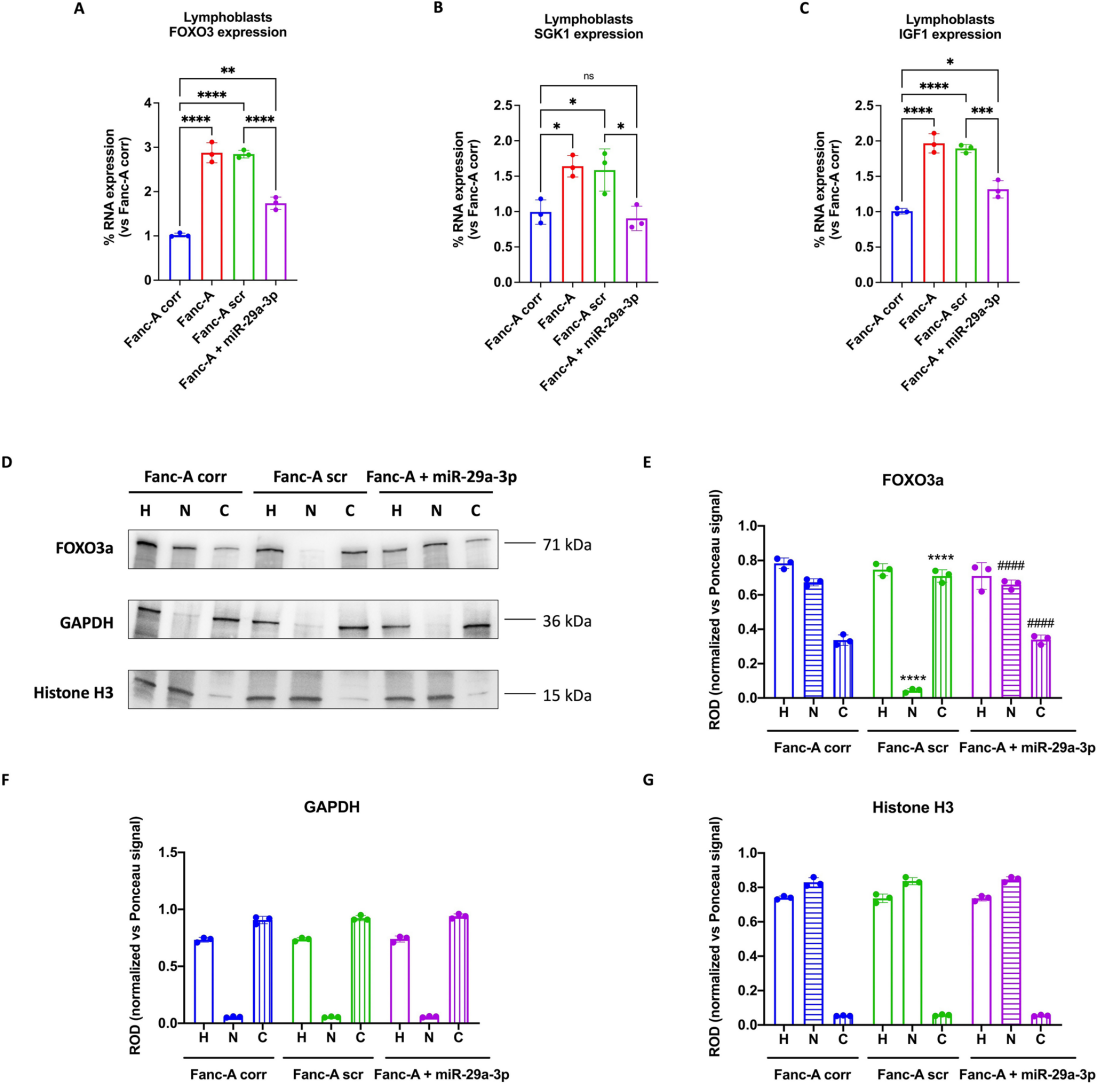
FOXO3, SGK1, and IGF1 expression and FOXO3a intracellular localization in Fanc-A lymphoblasts. In the top of the figure graphs show the comparison of FOXO3 (**A**), SGK1 (**B**), and IGF1 (**C**) expression in (i) Fanc-A cells corrected with the WT Fanc-A gene (Fanc-A corr), (ii) Fanc-A cells (Fanc-A), (iii) Fanc-A cells transfected with a miRNA mimic negative control for 48 h (Fanc-A scr), and (iv) Fanc-A cells transfected with miR-29a-3p for 48 h (Fanc-A + miR-29a-3p). GAPDH was used as the reference control. Data are expressed as mean ± SD and are representative of three independent experiments (*n* = 3) To investigate the intracellular localization of FOXO3a, all analyses were conducted on cell homogenate (H), nuclear fraction (N), and cytoplasmic fraction (**C**) derived from Fanc-A corr, Fanc-A scr, and Fanc-A + miR-29a-3p. (**D**) Representative WB signals of FOXO3a, GAPDH (used as a cytoplasmic marker), and Histone H3 (used as a nuclear marker). The virtual absence of the GAPDH signal in the nuclear fraction and the Histone H3 signal in the cytoplasmic fraction demonstrates the correct separation of the two cellular fractions. (**E**) Densitometric analysis of the FOXO3a signal. (**F**) Densitometric analysis of the GAPDH signal. (**G**) Densitometric analysis of the histone H3 signal. Data in panels E-G are expressed as mean ± SD and are representative of three independent experiments (*n* = 3). *, **, ***, and **** indicate a significant difference for *p* < 0.05, 0.01, 0.001, and 0.0001, respectively. ns indicates a no-significant statistical difference

The data presented in Fig. 1B highlight that FOXO3, SGK1, and IGF1 are implicated in multiple pathways, including DNA damage response, mitochondrial activity regulation, oxidative stress management, and apoptosis, all hallmark features of FA pathology. Consequently, the expression of these genes was assessed in Fanc-A lymphoblasts (Fig. 3A-C) and Fanc-A fibroblasts (Fig. S3A-C). The results revealed significantly elevated expression levels for all three genes compared to their corrected counterparts. By contrast, in Fanc-A cells transfected with miR-29a-3p, the expression of the FOXO3, SGK1, and IGF1 genes decreased by about 40%, 34%, and 30%, respectively, compared to the FA cells, reaching a comparable level compared to control cells.

### Treatment with miR-29a-3p restores the nuclear translocation of FOXO3a in Fanc-A lymphoblasts

WB analysis of FOXO3a localization revealed that Fanc-A lymphoblasts predominantly expressed FOXO3a in the cytoplasmic fraction while Fanc-A corr cellsexhibited higher protein expression in the nucleus. Conversely, miR-29a-3p transfection in Fanc-A cells significantly promoted FOXO3a translocation to the nucleus, enhancing its transcriptional activity (Fig. 3D-G).

### The miR-29a-3p-induced nuclear translocation of FOXO3a depends on the modulation of its hyperphosphorylation through AKT and SGK1 pathways

**Fig. 4:**
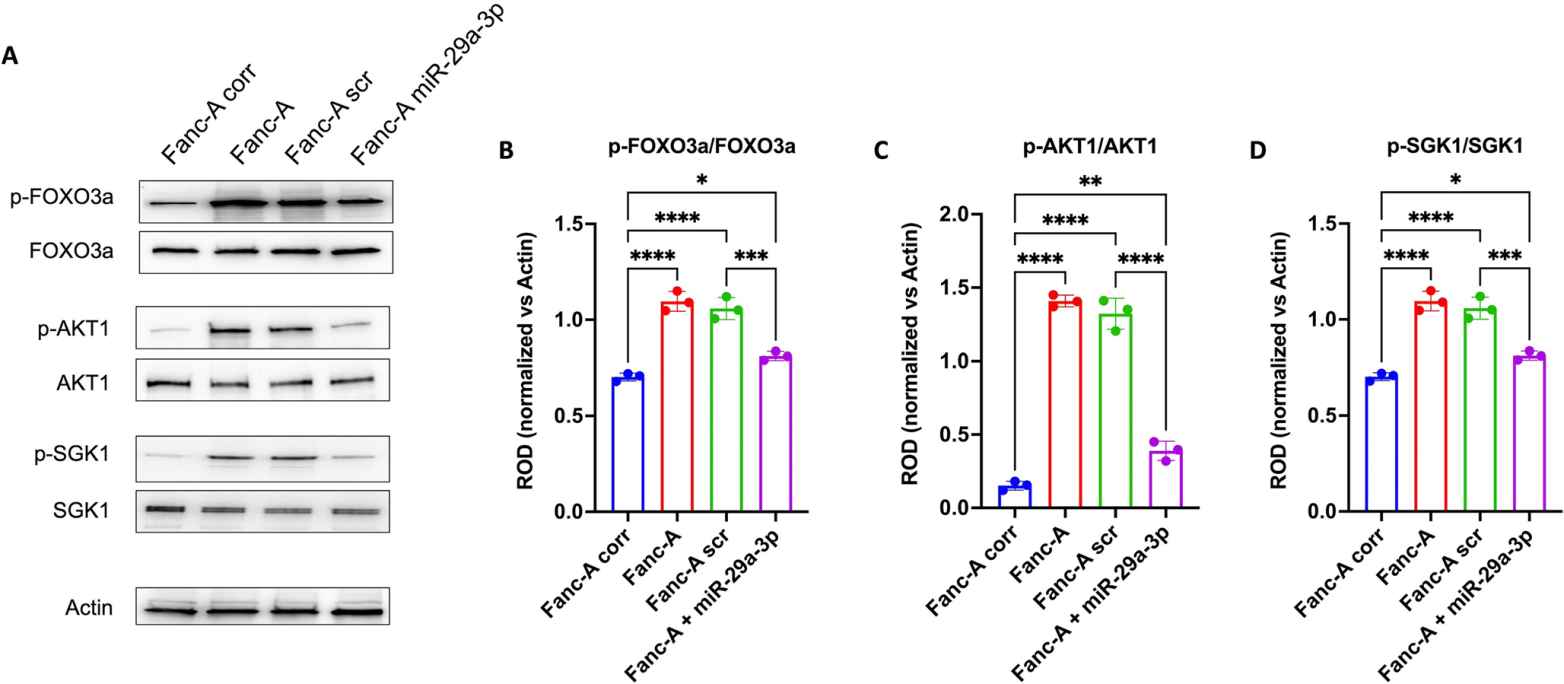
miR-29a-3p transfection modulates the FOXO3a, AKT, and SGK1 phosphorylation in Fanc-A lymphoblasts. All analyses were conducted on Fanc-A lymphoblasts corrected with the WT *Fanc-A* gene (Fanc-A corr), Fanc-A lymphoblasts (Fanc-A), Fanc-A lymphoblasts transfected with a miRNA mimic negative control for 48 h (Fanc-A scr), and Fanc-A lymphoblasts transfected with miR-29a-3p for 48 h (Fanc-A + miR-29a-3p) (**A**) Representative WB signals of: phospho-FOXO3a (Ser253); total FOXO3a; phospho-AKT (Ser473); total AKT; phospho-SGK1 (Ser422); total SGK1. Actin signal was used as housekeeping. (**B**) The ratio of phosphorylated and total forms of FOXO3a signals. (**C**) The ratio of phosphorylated and total forms of AKT signals. (D) The ratio of phosphorylated and total forms of SGK1 signals. Data in panels B, C, and D are expressed as mean ± SD and are representative of three independent experiments (*n* = 3). *, **, ***, and **** indicate a significant difference for *p* < 0.05, 0.01, 0.001, and 0.0001, respectively

FOXO3a migration from cytoplasm to nucleus depends on different post-transcriptional modifications [53–55], including the phosphorylation on Ser253 by AKT phosphorylated on Ser473, which is the principal post-translational modification that prevents FOXO3a entry into the nucleus [56, 57]. Therefore, the phosphorylation levels of these two proteins were evaluated by WB analysis. Our results show that Fanc-A lymphoblasts displayed higher phosphorylation of Ser253 p-FOXO3a (Fig. 4A and B) and Ser473 p-AKT (Fig. 4A and C) compared to Fanc-A corrected cells, which was reverted by miR-29a-3p treatment. Furthermore, since SGK1 shares the same phosphorylation site on FOXO3a as AKT [58, 59] when phosphorylated on Ser422, the expression of phosphorylated and total forms of SGK1 in Fanc-A cells has been evaluated, observing a hyperphosphorylation in Fanc-A cells compared to Fanc-A corr, which was reduced after miR-29a-3p transfection(Fig. 4A and D).

### miR-29a-3p and TGF-β pathway modulate each other

**Fig. 5:**
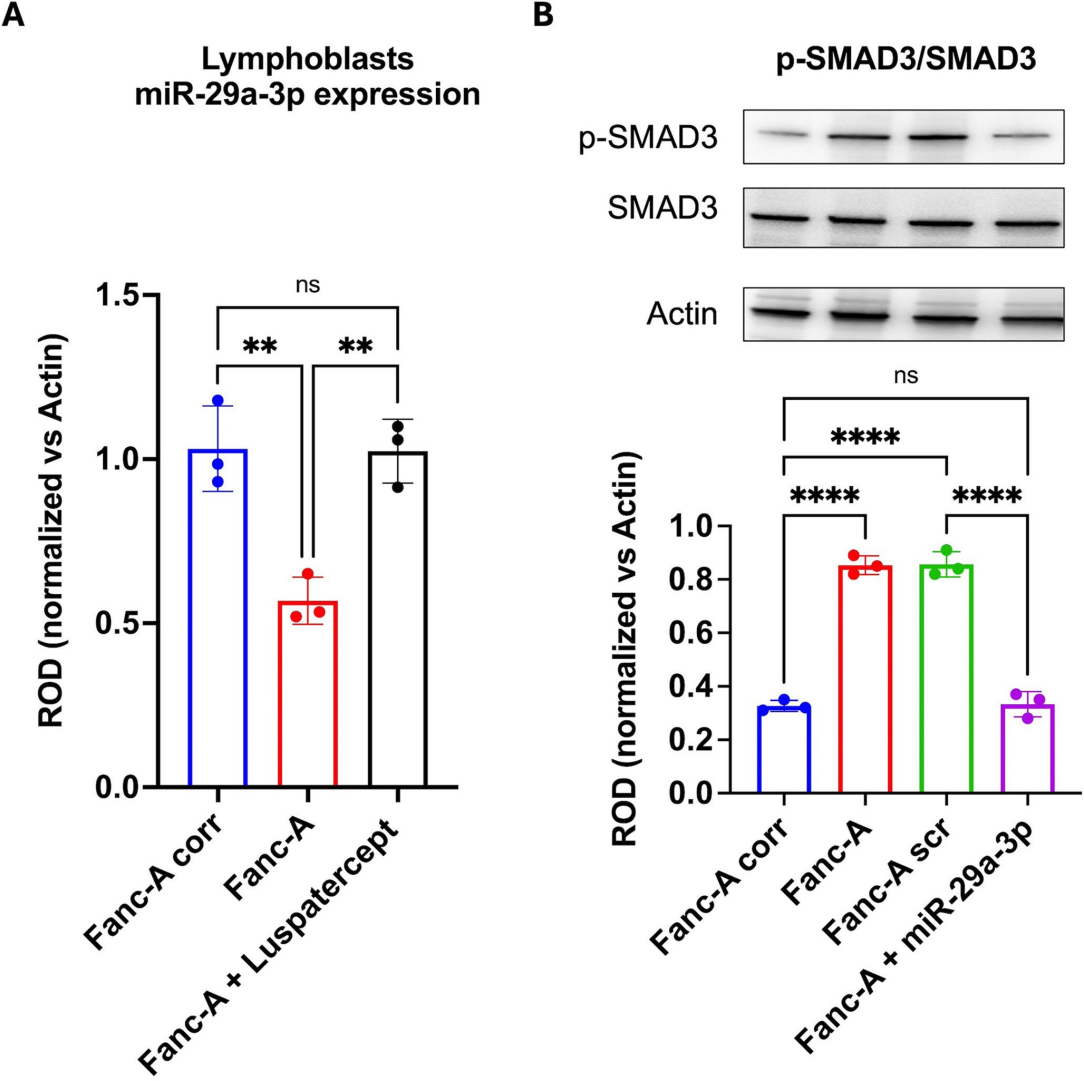
Reciprocal effect of SMAD3 inhibition and miR-29a-3p transfection in Fanc-A lymphoblasts. (**A**) miR-29a-3p expression in Fanc-A lymphoblasts corrected with the WT *Fanc-A* gene (Fanc-A corr), Fanc-A lymphoblasts (Fanc-A), Fanc-A lymphoblasts treated with Luspatercept (TGF-beta pathway inhibitor) for 48 h (Fanc-A + Luspatercept) (**B**) Representative WB signals of phospho-SMAD3 (Ser423/425), total SMAD3, and Actin (housekeeping protein) signals and relative ratio of phosphorylated and total forms of SMAD3 signals performed on Fanc-A corr, Fanc-A, Fanc-A lymphoblasts transfected with a miRNA mimic negative control for 48 h (Fanc-A scr), and Fanc-A lymphoblasts transfected with miR-29a-3p for 48 h (Fanc-A + miR-29a-3p). For both hystograms, data are expressed as mean ± SD and are representative of three independent experiments (*n* = 3).**, and **** indicate a significant difference for *p* < 0.01, and 0.0001, respectively. ns indicates a no-significant statistical difference

Since the miR-29a-3p expression is negatively regulated by the TGF-β pathway hyperactivation, its expression has been evaluated in the presence of Luspatercept, observing that treated FA lymphoblast increased the miR-29a-3p expression, reaching the level of control cells (Fig. 5A).

On the other hand, miR-29a-3p also affects TGF-β signaling, as miR-29a-3p transfection in Fanc-A lymphoblasts reduced SMAD3 hyperphosphorylation to levels comparable to those observed in corrected cells (Fig. 5B).

### Inhibition of TGF-βor IGF1 signaling reduces the oxidative damage and improves the oxidative phosphorylation in Fanc-A lymphoblasts

As shown in Fig. 5, the TGF-βsignal reduction leads to a recoveryof miR-29a-3p content in FA lymphoblasts, suggesting the evaluation of possible effects of Luspatercept on antioxidant defenses, DNA damage, and energy metabolism. Moreover, since FA cells displayed elevated IGF1 expression compared to the control, which, however, decreased after transfection with miR-29a-3p (Fig. 3C), the same investigation was considered appropriate for Klotho, an IGF1 signaling inhibitor [51].

**Fig. 6:**
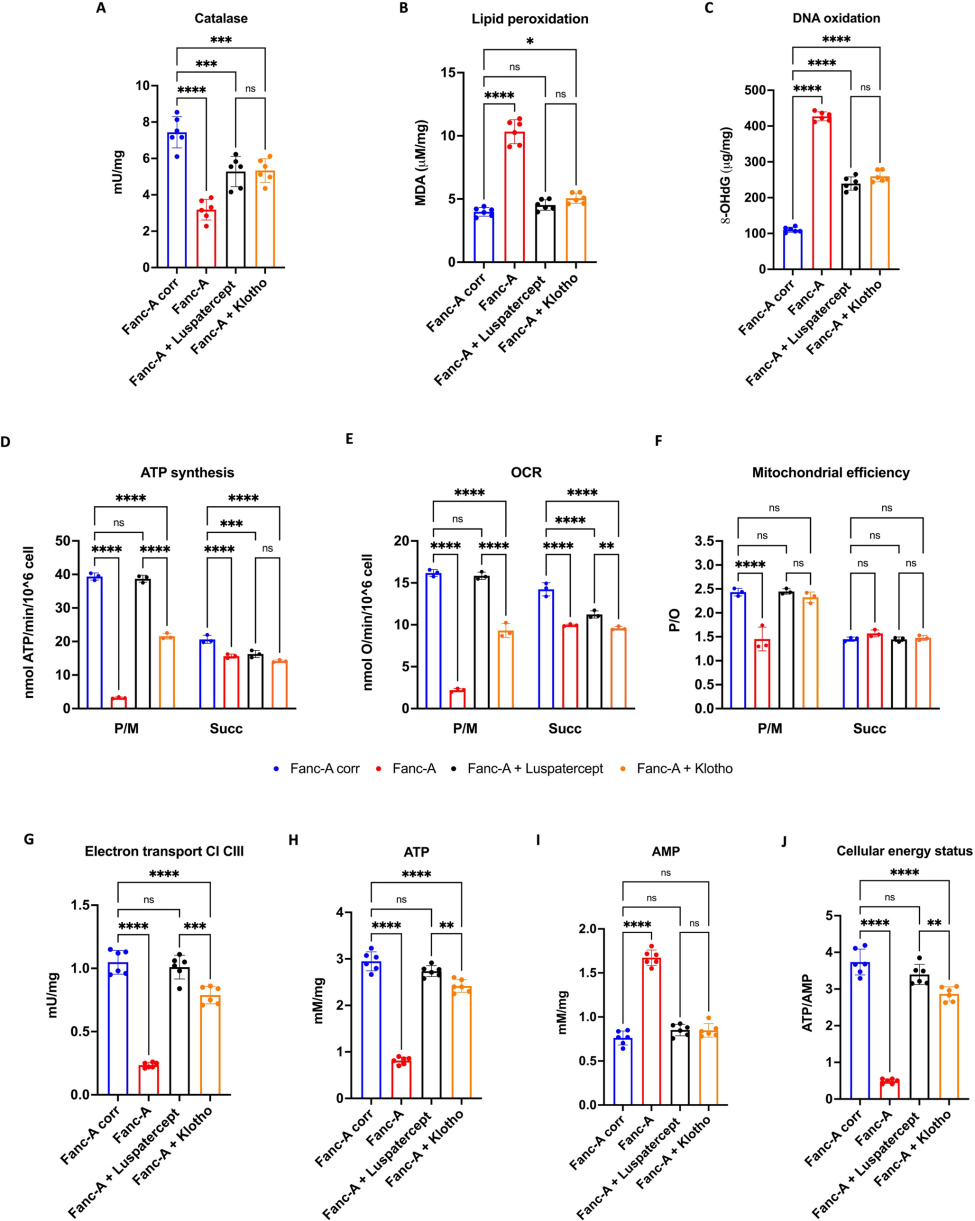
Antioxidant defenses, oxidative stress, and energy metabolism were modulated by Luspatercept or Klotho treatment in Fanc-A lymphoblasts. All analyses were conducted on Fanc-A lymphoblasts corrected with the WT *Fanc-A* gene (Fanc-A corr), Fanc-A lymphoblasts (Fanc-A), Fanc-A lymphoblasts treated with Luspatercept (TGF-β pathway inhibitor) for 48 h (Fanc-A + Luspatercept), and Fanc-A lymphoblasts treated with Klotho (IGF1 signaling inhibitor) for 48 h (Fanc-A + Klotho) (**A**) Catalase activity as an antioxidant defense marker. (**B**) Malondialdehyde (MDA) intracellular concentration, as a lipid peroxidation marker. (**C**) 8-hydroxy-2’-deoxyguanosine (8-OHdG) content as a DNA oxidation marker. (**D**) ATP synthesis through F_o_F_1_-ATP synthase. (**E**) Oxygen consumption rate (OCR). (**F**) P/O value, an OxPhos efficiency marker. For Panels D, E, and F, the analyses were conducted in the presence of pyruvate plus malate (P/M) or succinate (Succ) to induce the OxPhos pathways led by Complex I or Complex II, respectively. (**G**) Electron transfer between Complexes I and III. (**H**) Intracellular ATP content. (**I**) Intracellular AMP content. (**J**) Cellular energy status is obtained by calculating the ATP/AMP ratio Data are expressed as mean ± SD and are representative of three independent experiments (*n* = 3) for Panels D-F, and six independent experiments (*n* = 6) for Panels A-C and G-J. *, **, ***, and **** indicate a significant difference for *p* < 0.05, 0.01, 0.001, and 0.0001, respectively. ns indicates a no-significant statistical difference

The data show that both the inhibition of the TGF-β pathway and IGF1 signaling exhibit the same trend observed after transfecting FA cells with miR-29a-3p. In particular, Luspatercept and Klothotreatmentsincreased AO response (Fig. 6A), an evident reduction in oxidative stress accumulation(Fig. 6B-C), a recovery of mitochondrial function(Fig. 6D-G), and of cellular energy status (Fig. 6H-L).

### Luspatercept and Klotho treatments reduce the hyperphosphorylation of FOXO3a, SGK1, and AKT

**Fig. 7:**
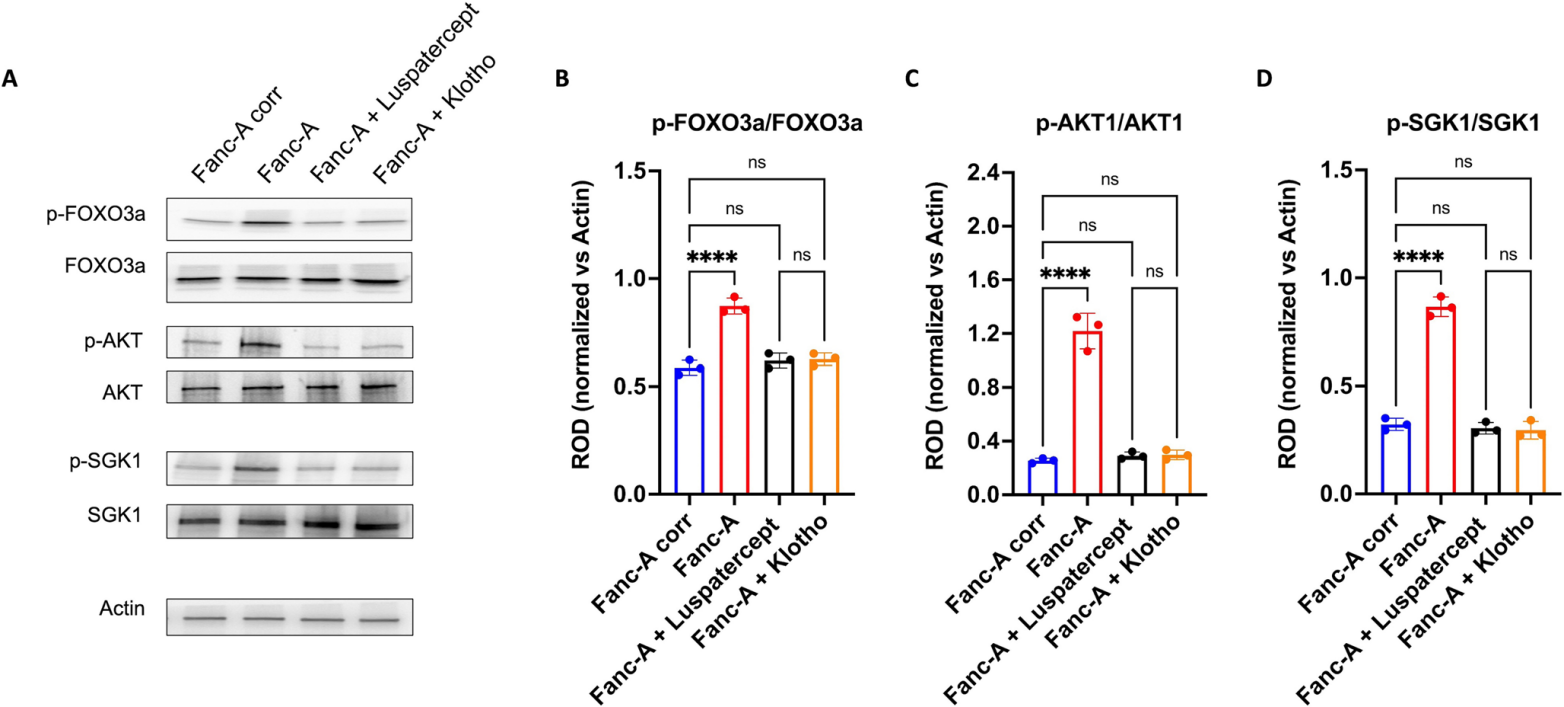
Luspatercept and Klotho treatments modulate the FOXO3a, AKT, and SGK1 phosphorylation in Fanc-A lymphoblasts. All analyses were conducted on Fanc-A lymphoblasts corrected with the WT *Fanc-A* gene (Fanc-A corr), Fanc-A lymphoblasts (Fanc-A), Fanc-A lymphoblasts treated with Luspatercept (TGF-beta pathway inhibitor) for 48 h (Fanc-A + Luspatercept), and Fanc-A lymphoblasts treated with Klotho (IGF1 signaling inhibitor) for 48 h (Fanc-A + Klotho) (**A**) Representative WB signals of: phospho-FOXO3a (Ser253); total FOXO3a; phosphor-AKT (Ser473); total AKT; phosphor-SGK1 (Ser422); total SGK1. Actin signal was used as housekeeping. (**B**) The ratio of phosphorylated and total forms of FOXO3a signals. (**C**) The ratio of phosphorylated and total forms of AKT signals. (**D**) The ratio of phosphorylated and total forms of SGK1 signals. Data in panels B, C, and D are expressed as mean ± SD and are representative of three independent experiments (*n* = 3). **** indicates a significant difference for *p* < 0.0001. ns indicates a no-significant statistical difference

Since Luspatercept and Klotho treatments modulate miR-29a-3p expression and downstream function, respectively, their effects on the hyperphosphorylation of FOXO3a, AKT, and IGF1 have been investigated. Data reported in Fig. 7 show that bothtreatments can reduce the phosphorylation level of miR-29a-3p targets, restoring levels similar to the control.

## Discussion

The data presented herein provide novel insights into the role of the miR-29a-3p and TGFβ axis in FA pathogenesis, particularly concerning mitochondrial dysfunction, redox balance, and DNA damage accumulation.

These findings suggest that, although the defect in DNA damage repair is the principal cause of FA, additional molecular mechanisms contribute to maintaining an altered cellular state, perpetuating a vicious cycle that exacerbates DNA damage over time.

Previous studies have highlighted the dysregulation of several miRNAs in FA cells [30–32], showing how FA cells are characterized by a different miRNA profile compared to healthy individuals and how BMT can partially revert this alteration, suggesting a key role of miRNAs in the pathogenesis of FA. However, to the best of our knowledge, this is the first study to demonstrate that the downregulation of miR-29a-3p impacts energetic and redox imbalances typical of FA cells. In detail, we report that the miR-29a-3p transfection improved OxPhos in terms of function and coupling and promoted the antioxidant enzyme expression and activity in FA lymphoblasts and fibroblasts. This enhancement of redox balance prevented membrane lipoperoxidation, as evidenced by reduced MDA levels, and mitigated DNA damage, as indicated by decreased 8-OHdG content and reduced H2AX phosphorylation. On the other hand, miR-29a-3p has emerged as a critical regulator, modulating the expression of genes involved in mitochondrial activity [41], redox balance [42], DNA damage [42], and cell proliferation [60].

To understand the mechanism underlying the influence of miR-29a-3p on the energy metabolism and redox balance of FA cells, we focused our attention on FOXO3a, a miR-29a-3p-targeted transcription factor playing a pivotal role in the mitochondrial metabolism modulation [41]. On the other hand, miR-29a-3p appears essential for the self-renewal of hematopoietic stem cells just by controlling mitochondrial function [61]. Our data show that FA cells displayed a threefold higher expression of the FOXO3 gene compared to control cells. This elevated expression in FA cells could explain the metabolic alterations characterizing FA cells as FOXO3a activation causes a reduction in mitochondrial DNA copy number, mitochondrial protein expression, respiratory complexes, and mitochondrial respiratory activity [62]. This hypothesis is confirmed by the recovery of OxPhos activity and efficiency as well as the cellular energy status improvement observed after the reduction of FOXO3 gene expression following the transfection with miR-29a-3p both in FA lymphoblasts and fibroblasts. In addition, behind the FOXO3 gene expression, it is necessary to consider that FOXO3a protein functiondepends on its cellular localization, which is regulated by various post-translational modifications; for example, FOXO3a nuclear translocation is promoted by the macrophage stimulating 1 (MST1)-induced phosphorylation while it is inhibited by thephosphorylation on Thr32 and Ser253 by Ser473-phosphorylated AKT [58]. Since AKT hyperphosphorylation is a hallmark of FA cells [63], it is quite surprising to observe that, in FA lymphoblasts, FOXO3a appears hyperphosphorylated at Ser253 and accumulates in the cytoplasmic fraction. Interestingly, both FOXO3a and AKT hyperphosphorylation were reduced in cells transfected with miR-29a-3p, leading to an increase in FOXO3a levels in the nuclear fraction, suggesting that the AKT pathway is also regulated by miR-29a-3p [64]. Indeed, a recent study by Pang et al. proposes that the FOXO3a localization in nuclei depends on the mono-ubiquitination of FANCD2 and is independent of AKT phosphorylation. However, this discrepancy suggests that the regulation of FOXO3a nuclear localization is highly complex and involves additional post-translational modifications [65].

Since FOXO3a also promotes ROS detoxification by increasingcellular antioxidant defense [66] and genome stability [52], it is plausible to speculate that the miR-29a-3p-induced FOXO3 gene and protein modulation also leadsto the increase in catalase activity and the decrease of the DNA damage accumulation observed in transfected FA lymphoblasts and fibroblasts.

**Fig. 8:**
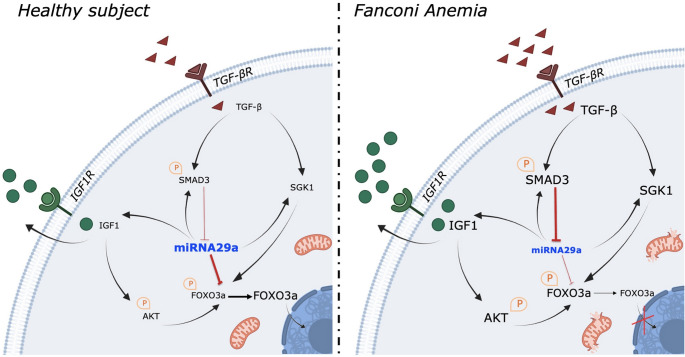
Role of miRN-29a-3p as a dynamic modulator of FA pathogenesis. The image show the role of miR-29a-3p in the modulation of FOXO3a, IGF1, and TGF-β signals in healthy cells (left) and FA cell (right).The font size is proportional to the expression level of the indicated protein

The SGK1, another miR-29a-3p target gene, also modulates FOXO3a phosphorylation and the consequent cellular localization. In detail, SGK1 phosphorylated on Ser422 leads to the FOXO3a negative modulation [58]. Regarding this, our data show that FA lymphoblasts are characterized by elevated SGK1 gene expression and hyperphosphorylation of the SGK1 protein, which return to levels similar to those of controls following transfection with miR-29a-3p. Therefore, these data suggest that miR-29a-3p transfection exerts a dual effect on FOXO3a: it acts as a direct modulator of its gene expression while also influencing its cellular localization and function through the regulation of AKT and SGK1, another target gene of miR-29a-3p. In other words, the reduction of miR-29a-3p triggers a cascade signal that facilitates the DNA damage, as resumed in Fig. 8 and by the following flow: Fanc-A gene KO **→ miR29a-3p↓ → TGF-β↑** (via SMAD3^P^) + SGK1^P^↑ + AKT^P^↑ + FOXO3^P^↑ → FOXO3a nuclear translocation↓ →Mitochondrial energy metabolism efficiency↓ and antioxidant defences↓ → oxidative stress↑ → DNA damage↑.

To investigate the mechanism underlying the downregulation of miR-29a-3p in FA cells, we focused on the TGF-β signaling, as this pathway is hyperactivated in FA cells [67]. In addition, the miR-29a-3p expression is modulated by TGF-β through the signal transducer SMAD3 [68]. Indeed, our data show that inhibition of TGF-β signaling following treatment with Luspatercept leads to an increased expression of miR-29a-3p in FA lymphoblasts, which, in turn, exerts positive effects on mitochondrial metabolism, cellular energy status, and the activation of antioxidant defenses. The same results were also observed in the presence of Klotho, an inhibitor of IGF1 signaling, which is an effector of the TGF-β pathway [69]. IGF1 expression is elevated in FA cells and is regulated by miR-29a-3p. This finding is particularly interesting given that FA patients are characterized by borderline hyperglycemia [70], which could lead to increasing in IGF1 signaling.

The inhibition of the TGF-β signal throughLuspatercept or Klotho also causes a reduction of DNA damage accumulation, as demonstrated by the decrement in 8-OHdG content and H2AX phosphorylation. On the other hand, in FA cells, the hyperactivation of TGF-β signaling promotes DNA repair via non-homologous end joining (NHEJ), an error-prone repair pathway that contributes to toxicity in FA hematopoietic stem cells [67]. Conversely, inhibition of the TGF-β pathway through silencing of the SMAD3 gene in FA mice and human cells modulates the expression of DNA repair genes in favor of homologous recombination (HR) over NHEJ, resulting in increased growth of hematopoietic progenitors and rescue of bone marrow failure [29].

In addition, TGF-β signaling inhibition plays a pivotal role in modulating the PI3K/AKT pathway [71, 72] also influencing FOXO3a, as demonstrated by the decreased phosphorylation of AKT, SGK1, and FOXO3a following treatment with Luspatercept and Klotho.

Interestingly, miR-29a-3p is also a modulator of the TGF-β pathway, as its transfection into FA cells reduces the phosphorylation of SMAD3, a key effector of the TGF-β pathway. In other words, the data suggest that miR-29a-3p and TGF-β are connected through a negative feedback loop that mutually regulates their expression.

Therefore, if increased miR-29a-3p expression can restore mitochondrial functionality and redox balance while reducing DNA damage accumulation in FA cells through the modulation of AKT, SGK1, and FOXO3a, it is plausible to hypothesize that thismodulation, in turn, reduces the release of pro-inflammatory cytokines, leading to a consequent decrease of the TGF-β signaling, further promoting miR-29a-3p expression. In other words, the mutual modulation of miR-29a-3p and TGF-β signaling can be resume in two flows that converge in the reduction of mitochondrial and DNA damage:

(A) **TGF-β↓** (via Luspatercept) **→ miR29a-3p↑** → SGK1^P^↓ + AKT^P^↓ + FOXO3^P^↓ → FOXO3a nuclear translocation↑ →Mitochondrial energy metabolism efficiency↑ and antioxidant defences↑ → oxidative stress↓ → DNA damage↓.
(B) **miR29a-3p↑ → TGF-β↓**(via SMAD3^P^) → SGK1^P^↓ + AKT^P^↓ + FOXO3^P^↓ → FOXO3a nuclear translocation↑ →Mitochondrial energy metabolism efficiency↑ and antioxidant defences↑ → oxidative stress↓ → DNA damage↓.

## Conclusions

The data presented in this study demonstrate the central role of miR-29a-3p and the TGF-β pathway in the pathogenesis of FA, as the overexpression of the former and the downregulation of the latter promote the recovery of metabolic functionality, the restoration of redox balance, and the reduction of DNA damage accumulation. These findings also suggest that the cellular dysfunctions characteristic of FA, which lead to DNA damage accumulation, depend on the activation of multiple, partially redundant signaling pathways (Fig. 8). Thus, although FA remains a DNA repair disorder, other molecular alterations—triggered by DNA damage but which, in turn, lead to further accumulation of DNA mutations—may contribute to disease pathogenesis, such as the enhancement of inflammatory and oxidative stress environments, representing potential molecular targets for new therapies. Consequently, any effective therapeutic approach should aim to modulate the FA cells alterations at multiple levels.

In addition, since impaired mitochondrial function, oxidative stress and pro-inflammatory state are functional defects known to be involved in the development of aplastic anemia and further neoplastic evolution, these data may represent an interesting model for studying the pathogenesis of inherited or acquired bone marrow failure syndromes.

## Supplementary Information

Below is the link to the electronic supplementary material.

ESM1 (DOCX 638 KB)

ESM2 (PDF 1.78 MB)

## Data Availability

Further information and requests for resources and reagents should be directed to and will be fulfilled by the lead contact, Dr. Enrico Cappelli (EnricoCappelli@gaslini.org). All data supporting the conclusions of this study can be found in the Article and Supplementary Material.

## References

[1] de Winter JP, Joenje H (2009) The genetic and molecularbasis of Fanconi anemia. Mutat Res 668:11–19. 10.1016/j.mrfmmm.2008.11.004 19061902 10.1016/j.mrfmmm.2008.11.004

[2] Svahn J, Dufour C (2011) Fanconi anemia - learning from children. Pediatr Rep 3(Suppl2):e8–e8. 10.4081/pr.2011.s2.e8 22053284 10.4081/pr.2011.s2.e8 PMC3206526

[3] Grompe M, D’Andrea A (2001) Fanconi anemia and DNA repair. Hum Mol Genet 10:2253–2259. 10.1093/hmg/10.20.2253 11673408 10.1093/hmg/10.20.2253

[4] Yang Y-G, Herceg Z, Nakanishi K et al (2005) The Fanconi anemia group A proteinmodulateshomologousrepair of DNA double-strand breaks in mammaliancells. Carcinogenesis 26:1731–1740. 10.1093/carcin/bgi134 15905196 10.1093/carcin/bgi134

[5] Ravera S, Dufour C, Degan P, Cappelli E (2018) Fanconi anemia: From DNA repair to metabolism. Eur J Hum Genet 26. 10.1038/s41431-017-0046-6 10.1038/s41431-017-0046-6 PMC5891494 29396564

[6] Cappelli E, Ravera S, Vaccaro D et al (2013) Mitochondrialrespiratorycomplex I defects in Fanconi anemia. Trends Mol Med19. 10.1016/j.molmed.2013.07.008 10.1016/j.molmed.2013.07.008 23932594

[7] Du W, Adam Z, Rani R et al (2008) Oxidative stress in Fanconi anemia hematopoiesis and diseaseprogression. Antioxid Redox Signal 10:1909–1921. 10.1089/ars.2008.2129 18627348 10.1089/ars.2008.2129 PMC2695607

[8] Li J, Sipple J, Maynard S et al (2012) Fanconi Anemia links reactiveoxygenspecies to insulinresistance and obesity. Antioxid Redox Signal 17:1083–1098. 10.1089/ars.2011.4417 22482891 10.1089/ars.2011.4417 PMC3423795

[9] Zhang X, Sejas DP, Qiu Y et al (2007) Inflammatory ROS promote and cooperate with the Fanconi anemia mutation for hematopoieticsenescence. J Cell Sci 120:1572–1583. 10.1242/jcs.003152 17405815 10.1242/jcs.003152 PMC2857731

[10] Vanderwerf SM, Svahn J, Olson S et al (2009) TLR8-dependent TNF-α overexpression in Fanconi anemia group C cells. Blood 114:5290–5298. 10.1182/BLOOD-2009-05-222414 19850743 10.1182/blood-2009-05-222414 PMC2796134

[11] Svahn J, Lanza T, Rathbun K et al (2015) P38 mitogen-activatedproteinkinaseinhibitionenhancesinvitroerythropoiesis of Fanconi anemia, complementation group A-deficientbonemarrowcells. 10.1016/j.exphem.2014.11.010. ExpHematol43:

[12] Dufour C, Corcione A, Svahn J et al (2003) TNF-alpha and IFN-gamma are overexpressed in the bone marrow of Fanconi anemia patients and TNF-alpha suppresseserythropoiesis in vitro. Blood 102:2053–2059. 10.1182/blood-2003-01-0114 12750172 10.1182/blood-2003-01-0114

[13] Korthof ET, Svahn J, Peffault de Latour R et al (2013) Immunologicalprofile of Fanconi anemia: a multicentricretrospectiveanalysis of 61 patients. Am J Hematol 88:472–476. 10.1002/ajh.23435 23483621 10.1002/ajh.23435

[14] Ravera S, Vaccaro D, Cuccarolo P et al (2013) Mitochondrialrespiratory chain complex i defects in Fanconi anemia complementation group A. Biochimie 95. 10.1016/j.biochi.2013.06.006 10.1016/j.biochi.2013.06.006 23791750

[15] Bottega R, Nicchia E, Cappelli E et al (2018) Hypomorphic FANCA mutations correlate with mild mitochondrial and clinical phenotype in Fanconi anemia. Haematologica 103. 10.3324/haematol.2017.176131 10.3324/haematol.2017.176131 PMC5830397 29269525

[16] Cappelli E, Cuccarolo P, Stroppiana G et al (2017) Defects in mitochondrialenergeticfunctioncompels Fanconi anaemiacells to glycolyticmetabolism. BiochimBiophys Acta Mol. 10.1016/j.bbadis.2017.03.008. BasisDis1863:

[17] Cappelli E, Degan P, Bruno S et al (2020) The passage from bone Marrowniche to bloodstream triggers the metabolic impairment in Fanconi Anemia mononuclearcells. Redox Biol 36. 10.1016/j.redox.2020.101618 10.1016/j.redox.2020.101618 PMC7327247 32863220

[18] Lyakhovich A (2013) Damagedmitochondria and overproduction of ROS in Fanconi anemia cells. Rare Dis 1:e24048–e24048. 10.4161/rdis.24048 25002988 10.4161/rdis.24048 PMC3915560

[19] Pagano G, Degan P, D’Ischia M et al (2005) Oxidative stress as a multiple effector in Fanconi anaemia clinical phenotype. Eur J Haematol 75:93–100. 10.1111/J.1600-0609.2005.00507.X 16000125 10.1111/j.1600-0609.2005.00507.x

[20] Cappelli E, Bertola N, Bruno S et al (2021) A multidrugapproach to modulate the mitochondrialmetabolism impairment and relative oxidative stress in Fanconi Anemia complementation group A. Metabolites 12:6. 10.3390/metabo12010006 35050128 10.3390/metabo12010006 PMC8777953

[21] Bertola N, Regis S, Bruno S et al (2023) Effects of deacetylaseinhibition on the activation of the antioxidantresponse and aerobicmetabolism in cellular models of Fanconi Anemia. Antioxid (Basel) 12. 10.3390/ANTIOX12051100 10.3390/antiox12051100 PMC10215951 37237966

[22] Pagano G, Youssoufian H (2003) Fanconi anaemiaproteins: major roles in cellprotectionagainstoxidativedamage. BioEssays 25:589–595. 10.1002/bies.10283 12766948 10.1002/bies.10283

[23] Kumari U, YaJun W, HuatBay B, Lyakhovich A (2014) Evidence of mitochondrialdysfunction and impaired ROS detoxifyingmachinery in Fanconi anemia cells. Oncogene 33:165–172. 10.1038/onc.2012.583 23318445 10.1038/onc.2012.583

[24] Degan P, Bonassi S, De Caterina M et al (1995) In vivo accumulation of 8-hydroxy-2’-deoxyguanosine in DNA correlates with release of reactiveoxygenspecies in fanconi’sanaemia families. Carcinogenesis 16:735–741 7728950 10.1093/carcin/16.4.735

[25] Brosh RM, Bellani M, Liu Y, Seidman MM (2017) Fanconi anemia: a DNA repair disorder characterized by accelerateddecline of the hematopoietic stem cell compartment and other features of aging. Ageing Res Rev 33:67. 10.1016/J.ARR.2016.05.005 27223997 10.1016/j.arr.2016.05.005 PMC5114166

[26] Helbling-Leclerc A, Garcin C, Rosselli F (2021) Beyond DNA repair and chromosomeinstability—Fanconi anaemiaas a cellularsenescence-associatedsyndrome. Cell Death Differ 28:1159. 10.1038/S41418-021-00764-5 33723374 10.1038/s41418-021-00764-5 PMC8026967

[27] Landelouci K, Sinha S, Pépin G (2022) Type-I interferon signaling in Fanconi Anemia. Front Cell InfectMicrobiol 12:820273. 10.3389/FCIMB.2022.820273/BIBTEX 10.3389/fcimb.2022.820273 PMC8859461 35198459

[28] Wang J, Erlacher M, Fernandez-Orth J (2022) The role of inflammation in hematopoiesis and bone marrowfailure: what can welearn from mouse models? Front Immunol 13. 10.3389/FIMMU.2022.951937 10.3389/fimmu.2022.951937 PMC9403273 36032161

[29] Zhang H, Kozono DE, O’Connor KW et al (2016) TGF-β Inhibition rescues hematopoietic stem cell defects and bone marrowfailure in Fanconi Anemia. Cell Stem Cell 18:668. 10.1016/J.STEM.2016.03.002 27053300 10.1016/j.stem.2016.03.002 PMC4860147

[30] Degan P, Cappelli E, Longobardi M et al (2019) A global MicroRNA profile in Fanconi anemia: A pilot study. Metab Syndr Relat Disord 17. 10.1089/met.2018.0085 10.1089/met.2018.0085 30376422

[31] Cappelli E, Ravera S, Bertola N et al (2024) Advanced analysis and validation of a microRNA signature for Fanconi Anemia. Genes 15(820–15):820. 10.3390/GENES15070820 39062599 10.3390/genes15070820 PMC11276059

[32] Cagnan I, Keles M, Keskus AG et al (2022) Global MiRNA expression of bone marrowmesenchymal stem/stromalcellsderived from Fanconi anemia patients. Hum Cell 35:111–124. 10.1007/S13577-021-00626-9/FIGURES/6 34792755 10.1007/s13577-021-00626-9

[33] Alizadeh M, Safarzadeh A, Beyranvand F et al (2019) The potentialrole of miR-29 in health and cancerdiagnosis, prognosis, and therapy. J Cell Physiol 234:19280–19297. 10.1002/JCP.28607 30950056 10.1002/jcp.28607

[34] Kwon JJ, Factora TD, Dey S, Kota J (2018) A systematic review of miR-29 in Cancer. Mol TherOncolytics 12:173. 10.1016/J.OMTO.2018.12.011 10.1016/j.omto.2018.12.011 PMC6369137 30788428

[35] Smyth A, Callaghan B, Willoughby CE, O’Brien C (2022) The role of miR-29 family in TGF-β drivenfibrosis in glaucomatousopticneuropathy. Int J Mol Sci 2022 23:10216. 10.3390/IJMS231810216 10.3390/ijms231810216 PMC9499597 36142127

[36] Wang M, Huo Z, He X et al (2023) The role of MiR-29 in the mechanism of fibrosis. Mini-Reviews MedicinalChemistry 23:1846–1858. 10.2174/1389557523666230328125031/CITE/REFWORKS 10.2174/1389557523666230328125031 37018517

[37] Pathania AS, Chava H, Chaturvedi NK et al (2024) The miR-29 family facilitates the activation of NK-cell immune responses by targeting the B7-H3 immune checkpoint in neuroblastoma. Cell Death &Disease 2024 15(6 15):1–16. 10.1038/s41419-024-06791-7 10.1038/s41419-024-06791-7 PMC11189583 38890285

[38] Yao XC, Wu JJ, Yuan ST, Yuan FL (2025) Recent insights and Perspectivesinto the role of the miRNA-29 family in innate immunity (Review). Int J Mol Med 55:1–12. 10.3892/IJMM.2025.5494/HTML 39886977 10.3892/ijmm.2025.5494 PMC11781520

[39] Yan B, Guo Q, Fu FJ et al (2015) The role of miR-29b in cancer: regulation, function, and signaling. Onco Targets Ther 8:539–548. 10.2147/OTT.S75899 25767398 10.2147/OTT.S75899 PMC4354468

[40] Amodio N, Rossi M, Raimondi L et al (2015) miR-29s: a family of epi-miRNAs with therapeuticimplications in hematologicmalignancies. Oncotarget 6:12837. 10.18632/ONCOTARGET.3805 25968566 10.18632/oncotarget.3805 PMC4536984

[41] Dalgaard LT, Sørensen AE, Hardikar AA, Joglekar MV (2022) The microRNA-29 family: role in metabolism and metabolicdisease. Am J Physiol Cell Physiol 323:C367–C377. 10.1152/AJPCELL.00051.2022/ASSET/IMAGES/LARGE/AJPCELL.00051.2022_F003.JPEG. 35704699 10.1152/ajpcell.00051.2022

[42] Jung Ydeun, Park SK, Kang D et al (2020) Epigeneticregulation of miR-29a/miR-30c/DNMT3A axis controls SOD2 and mitochondrialoxidative stress in human mesenchymal stem cells. Redox Biol 37:101716. 10.1016/J.REDOX.2020.101716 32961441 10.1016/j.redox.2020.101716 PMC7509080

[43] Yang YL, Kuo HC, Wang FS, Huang YH (2019) MicroRNA-29a disrupts DNMT3b to AmeliorateDiet-Induced Non-AlcoholicSteatohepatitis in mice. Int J Mol Sci 2019 20:1499. 10.3390/IJMS20061499 10.3390/ijms20061499 PMC6471363 30917489

[44] Hu W, Dooley J, Chung SS et al (2015) miR-29a maintains mouse hematopoietic stem cell self-renewal by regulating Dnmt3a. Blood 125:2206–2216. 10.1182/BLOOD-2014-06-585273 25634742 10.1182/blood-2014-06-585273 PMC4383797

[45] Xu X, Hong P, Wang Z et al (2021) MicroRNAs in TransformingGrowthFactor-Beta signaling pathway associated with fibrosisinvolvingdifferent systems of the human body. Front Mol Biosci 8:707461. 10.3389/FMOLB.2021.707461/BIBTEX 34381815 10.3389/fmolb.2021.707461 PMC8350386

[46] Zhang H, Kozono D, O’Connor K et al (2014) Bone marrowfailure in Fanconi Anemia from hyperactive TGF-β signaling. Blood 124:356. 10.1182/BLOOD.V124.21.356.356

[47] Liu Y, Bin, Wang Y, Zhang M, De et al (2020) MicroRNA-29a functionsas a tumor suppressorthrough targeting STAT3 in laryngealsquamouscell carcinoma. Exp Mol Pathol 116:104521. 10.1016/J.YEXMP.2020.104521 32858006 10.1016/j.yexmp.2020.104521

[48] Kutler DI, Auerbach AD, Satagopan J et al (2003) High incidence of head and neck squamouscell carcinoma in patients with Fanconi anemia. Arch Otolaryngol Head Neck Surg 129:106–112. 10.1001/ARCHOTOL.129.1.106 12525204 10.1001/archotol.129.1.106

[49] Bertola N, Bruno S, Capanni C et al (2023) AlteredMitochondrial dynamic in lymphoblasts and fibroblastsmutated for FANCA-A gene: the central role of DRP1. Int J Mol Sci 2023 24:6557. 10.3390/IJMS24076557 10.3390/ijms24076557 PMC10094900 37047537

[50] Hatzimichael E, Timotheatou D, Koumpis E et al (2022) Luspatercept: a Nnew Tool for the treatment of Anemia related to β-Thalassemia, MyelodysplasticSyndromes and PrimaryMyelofibrosis. Diseases 10:85. 10.3390/DISEASES10040085 36278584 10.3390/diseases10040085 PMC9624301

[51] Olejnik A, Radajewska A, Krzywonos-Zawadzka A, Bil-Lula I (2023) Klothoinhibits IGF1R/PI3K/AKT signalling pathway and protects the heart from oxidative stress during ischemia/reperfusioninjury. Sci Rep 2023 13(1):1–15. 10.1038/s41598-023-47686-5 10.1038/s41598-023-47686-5 PMC10662387 37985893

[52] Fasano C, Disciglio V, Bertora S et al (2019) FOXO3a from the Nucleus to the Mitochondria: A Round Trip in Cellular Stress Response. Cells 8:1110. 10.3390/CELLS8091110 31546924 10.3390/cells8091110 PMC6769815

[53] Skurk C, Maatz H, Kim HS et al (2004) The Akt-regulatedForkheadTranscriptionFactor FOXO3a controls endothelial cell viabilitythroughmodulation of the Caspase-8 inhibitor FLIP. J BiologicalChemistry 279:1513–1525. 10.1074/JBC.M304736200 10.1074/jbc.M304736200 14551207

[54] Boccitto M, Kalb G R (2011) Regulation of Foxo-dependenttranscription by post-translationalmodifications. Curr Drug Targets 12:1303–1310. 10.2174/138945011796150316 21443461 10.2174/138945011796150316 PMC3794852

[55] Liu Y, Ao X, Ding W et al (2018) Critical role of FOXO3a in carcinogenesis. Mol Cancer 2018 17(1):1–12. 10.1186/S12943-018-0856-3 10.1186/s12943-018-0856-3 PMC6060507 30045773

[56] Feehan RP, Shantz LM (2016) Negative regulation of the FOXO3a transcriptionfactor by mTORC2 induces a pro-survival response following exposure to ultraviolet-B irradiation. Cell Signal 28:798. 10.1016/J.CELLSIG.2016.03.013 27058291 10.1016/j.cellsig.2016.03.013 PMC4899167

[57] Brunet A, Bonni A, Zigmond MJ et al (1999) Aktpromotescell survival by phosphorylating and inhibiting a forkheadtranscriptionfactor. Cell 96:857–868. 10.1016/S0092-8674(00)80595-4 10102273 10.1016/s0092-8674(00)80595-4

[58] Wang X, Hu S, Liu L (2017) Phosphorylation and acetylationmodifications of FOXO3a: independently or synergistically? OncolLett 13:2867–2872. 10.3892/OL.2017.5851/HTML 10.3892/ol.2017.5851 PMC5431355 28521392

[59] Brunet A, Park J, Tran H et al (2001) Proteinkinase SGK mediates survival signals by phosphorylating the forkheadtranscriptionfactor FKHRL1 (FOXO3a). Mol Cell Biol 21:952–965. 10.1128/MCB.21.3.952-965.2001 11154281 10.1128/MCB.21.3.952-965.2001 PMC86685

[60] Xiao Z, Wang Y, Ding H (2019) XPD suppressescellproliferation and migration via miR-29a-3p-Mdm2/PDGF-B axis in HCC. Cell Biosci 9:1–12. 10.1186/S13578-018-0269-4/FIGURES/8 30627419 10.1186/s13578-018-0269-4 PMC6321695

[61] Hu W, Dooley J, Chung SS et al (2015) miR-29a maintains mouse hematopoietic stem cell self-renewal by regulating Dnmt3a. Blood 125:2206. 10.1182/BLOOD-2014-06-585273 25634742 10.1182/blood-2014-06-585273 PMC4383797

[62] Ferber EC, Peck B, Delpuech O et al (2011) FOXO3a regulatesreactiveoxygenmetabolism by inhibitingmitochondrial gene expression. Cell Death Diff 2012 19:968–979. 10.1038/cdd.2011.179 10.1038/cdd.2011.179 PMC3354049 22139133

[63] Ibáñez A, Río P, Casado JA et al (2009) Elevatedlevels of IL-1beta in Fanconi anaemia group A patients due to a constitutivelyactivephosphoinositide 3-kinase-Akt pathway are capable of promotingtumourcellproliferation. Biochem J 422:161–170. 10.1042/BJ20082118 19473116 10.1042/BJ20082118

[64] Li X, Li J, Wilson A et al (2015) Fancd2 isrequired for nuclearretention of Foxo3a in hematopoietic stem cellmaintenance. J BiolChem 290:2715–2727. 10.1074/JBC.M114.619536 10.1074/jbc.M114.619536 PMC4317007 25505262

[65] Wang Z, Yu T, Huang P (2016) Post-translationalmodifications of FOXO family proteins (Review). Mol Med Rep 14:4931–4941. 10.3892/MMR.2016.5867/HTML 27779663 10.3892/mmr.2016.5867

[66] Martins R, Lithgow GJ, Link W (2016) Long live FOXO: unraveling the role of FOXO proteins in aging and longevity. Aging Cell 15:196–207. 10.1111/ACEL.12427 26643314 10.1111/acel.12427 PMC4783344

[67] Rodríguez A, Epperly M, Filiatrault J et al (2022) TGFβ pathway isrequired for viablegestation of Fanconi anemia embryos. PLoS Genet 18:e1010459. 10.1371/JOURNAL.PGEN.1010459 36441774 10.1371/journal.pgen.1010459 PMC9731498

[68] Blahna MT, Hata A (2012) Smad-mediatedregulation of MicroRNA biosynthesis. FEBS Lett 586:1906–1912. 10.1016/J.FEBSLET.2012.01.041 22306316 10.1016/j.febslet.2012.01.041 PMC4429768

[69] Danielpour D, Song K (2006) Cross-talk between IGF-I and TGF-beta signaling pathways. CytokineGrowthFactorRev 17:59–74. 10.1016/J.CYTOGFR.2005.09.007 10.1016/j.cytogfr.2005.09.007 16297654

[70] Petryk A, Shankar RK, Giri N et al (2015) Endocrine disorders in Fanconi anemia: recommendations for screening and treatment. J ClinEndocrinolMetab 100:803. 10.1210/JC.2014-4357 10.1210/jc.2014-4357 PMC4333044 25575015

[71] Suwanabol PA, Seedial SM, Zhang F et al (2012) TGF-β and Smad3 modulate PI3K/Aktsignaling pathway in vascularsmooth muscle cells. Am J Physiol Heart CircPhysiol 302:H2211. 10.1152/AJPHEART.00966.2011 10.1152/ajpheart.00966.2011 PMC3378292 22447946

[72] Zhang L, Zhou F, tenDijke P (2013) Signaling interplaybetweentransforminggrowthfactor-β receptor and PI3K/AKT pathways in cancer. TrendsBiochem Sci 38:612–620. 10.1016/J.TIBS.2013.10.001 10.1016/j.tibs.2013.10.001 24239264

