# Supplementary material for: miR-29a-3p and TGF-β Axis in Fanconi anemia: mechanisms driving metabolic dysfunction and genome stability
Source: Cell Mol Life Sci. 2025 Jun 25;82(1):255. doi: 10.1007/s00018-025-05775-w (PMC12187631; doi:10.1007/s00018-025-05775-w)
Supplement: Supplementary file 1 — (DOCX 638 KB) [file 18_2025_5775_MOESM1_ESM.docx]

**SUPPLEMENTARY MATERIAL**

**Figure Supplementary 1 (S1)**

**
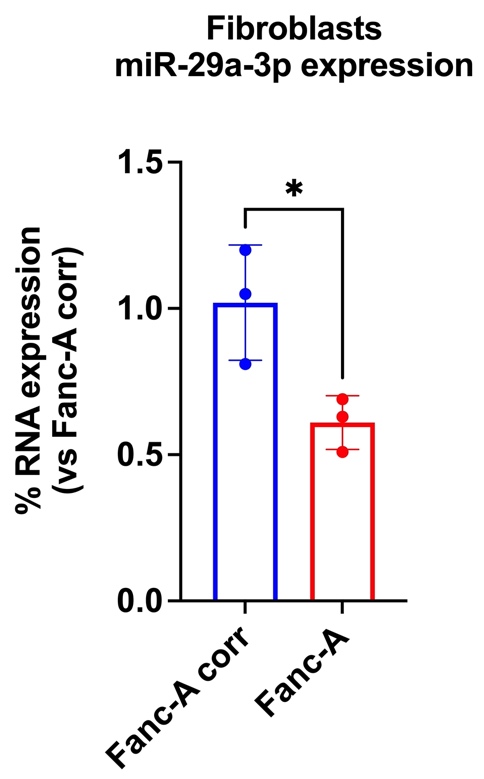
**

Figure S1. **miR-29a-3p expression in Fanc-A fibroblasts.**The graph shows the comparison of miR-29a-3p expression between Fanc-A fibroblasts corrected with the WT *Fanc-A* gene (Fanc-A corr) and Fanc-A fibroblasts (Fanc-A). RNU44 was used as a reference control. Data are expressed as mean ± SD and are representative of three independent experiments (n = 3). * indicates a significant difference for p <0.01.

**Figure Supplementary 2 (S2)**


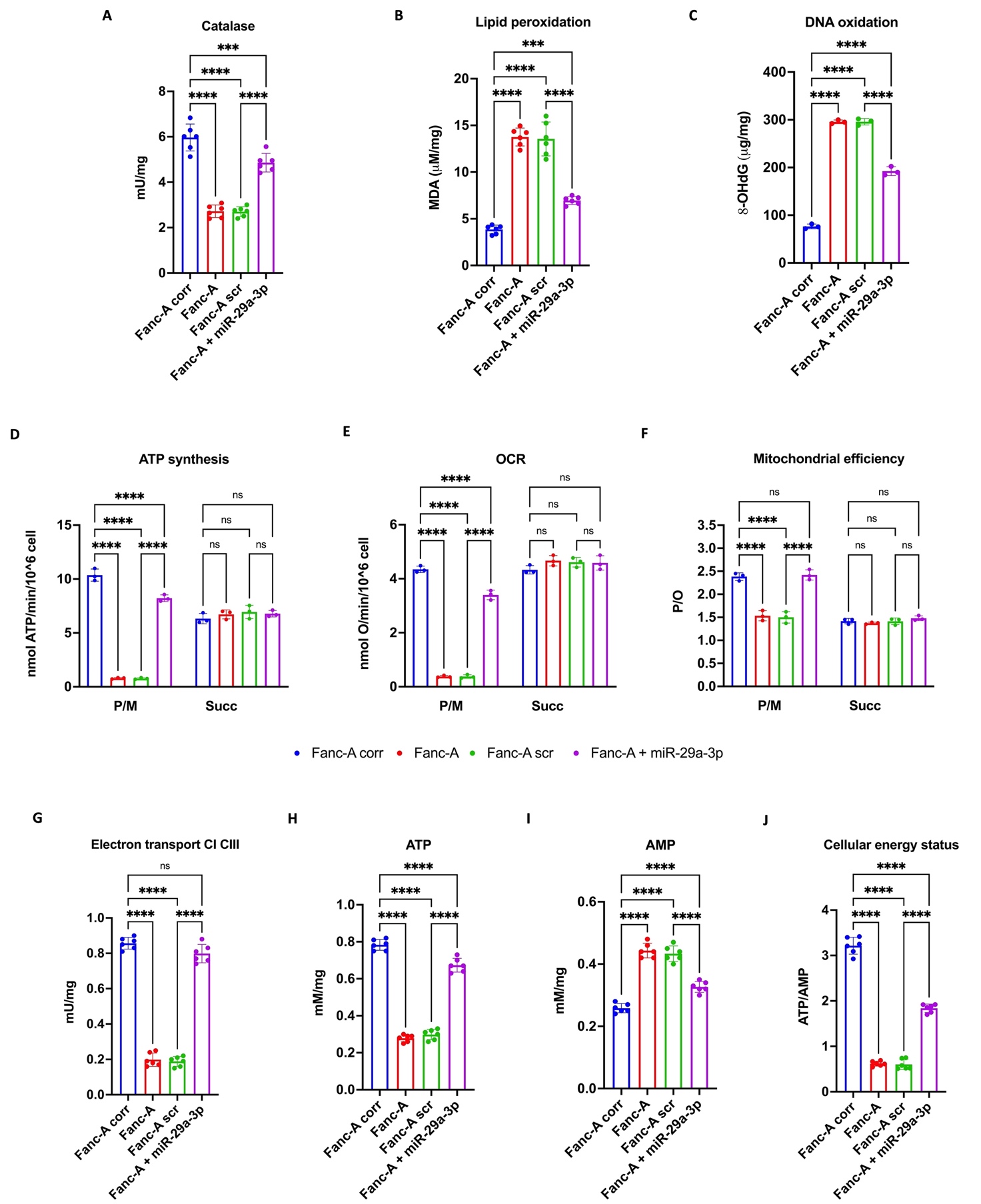


Figure S2. **Antioxidant defenses, oxidative stress, and energy metabolism were modulated by miR-29a-3p expression in Fanc-A fibroblasts.**

All analyses were conducted on Fanc-A fibroblasts corrected with the WT *Fanc-A* gene (Fanc-A corr), Fanc-A fibroblasts (Fanc-A), Fanc-A fibroblasts transfected with empty vector for 48h (Fanc-A scr), and Fanc-A fibroblasts transfected with miR-29a-3p for 48h (Fanc-A + miR-29a-3p).

(A) Catalase activity, as an antioxidant defenses marker. (B) Malondialdehyde (MDA) intracellular concentration, as a lipid peroxidation marker. (C) 8-hydroxy-2’-deoxyguanosine (8-OHdG) content, as a DNA oxidation marker. (D) ATP synthesis through F_o_F1-ATP synthase. (E) Oxygen consumption rate (OCR). (F) P/O value, an OxPhos efficiency marker. For Panels D, E, and F, the analyses were conducted in the presence of pyruvate plus malate (P/M) or succinate (Succ) to induce the OxPhos pathways led by Complex I or Complex II, respectively. (G) Electron transfer between Complexes I and III. (H) Intracellular ATP content. (I) Intracellular AMP content. (J) Cellular energy status is obtained by calculating the ATP/AMP ratio.

Data are expressed as mean ± SD and are representative of three independent experiments (n = 3) for Panels C-F and six independent experiments for Panels A-B and G-J (n = 6). ***, and **** indicate a significant difference for p <0.001, and 0.0001, respectively. ns indicates a no-significant statistical difference.

**Figure Supplementary 3 (S3)**


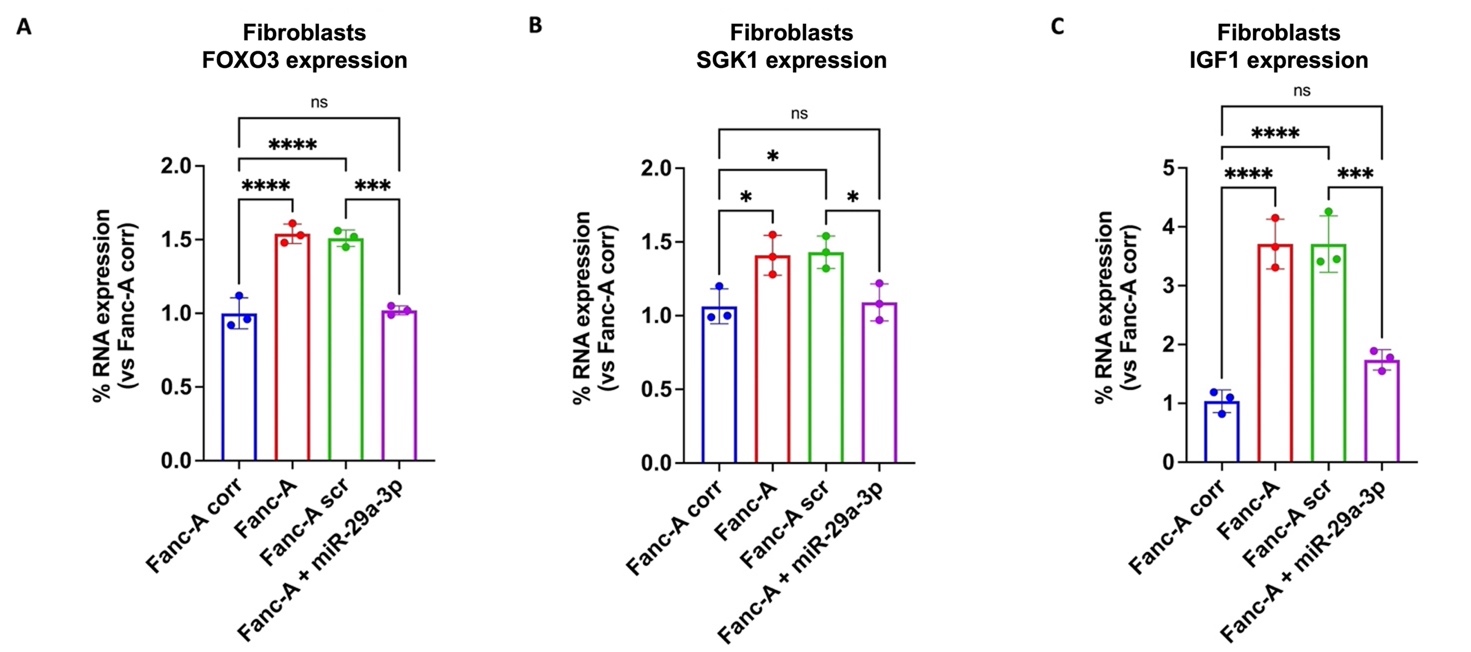


Figure S3. **FOXO3, SGK1, and IGF1 expression in Fanc-A fibroblasts**. Graphs show the comparison of FOXO3 (A), SGK1 (B), and IGF1 (C) expression in (i) Fanc-A cells corrected with the wt Fanc-A gene (Fanc-A corr), (ii) Fanc-A cells (Fanc-A), (iii) Fanc-A cells transfected with a miRNA mimic negative control for 48h (Fanc-A scr), and (iv) Fanc-A cells transfected with miR-29a-3p for 48h (Fanc-A + miR-29a-3p). GAPDH was used as the reference control. Data are expressed as mean ± SD and are representative of three independent experiments (n = 3). *, ***, and **** indicate a significant difference for p <0.05, 0.001, and 0.0001, respectively. ns indicates a no-significant statistical difference.

**Supplementary Table 1:** Ct standard deviation values of used reference genes among described treatments

| **Experiment** | **Reference gene** | **Ct standard deviation** |
| --- | --- | --- |
|  |  |  |
| Fig. 1A. miR-29a-3p in Fanconi lymphoblasts | RNU44 | 0.313 |
| Fig. S1. miR-29a-3p in Fanconi fibroblasts | RNU44 | 0.791 |
| Fig. 3A. FOXO3 in miR-29a-3p-transfected lymphoblasts | GAPDH | 0.532 |
| Fig. 3B. SGK1 in miR-29a-3p-transfected lymphoblasts | GAPDH | 0.321 |
| Fig. 3C. IGF1 in miR-29a-3p-transfected lymphoblasts | GAPDH | 0.532 |
| Fig. S3A. FOXO3 in miR-29a-3p-transfected fibroblasts | GAPDH | 0.295 |
| Fig. S3B. SGK1 in miR-29a-3p-transfected fibroblasts | GAPDH | 0.560 |
| Fig. S3C. IGF1 in miR-29a-3p-transfected fibroblasts | GAPDH | 0.263 |
| Fig. 5A. miR-29a-3p in lymphoblasts treated with Luspatercept | RNU44 | 0.489 |
