# Supplementary figures and images for: miR-29a-3p and TGF-β Axis in Fanconi anemia: mechanisms driving metabolic dysfunction and genome stability
Source: Cell Mol Life Sci. 2025 Jun 25;82(1):255. doi: 10.1007/s00018-025-05775-w (PMC12187631; doi:10.1007/s00018-025-05775-w)

**Figure 3**

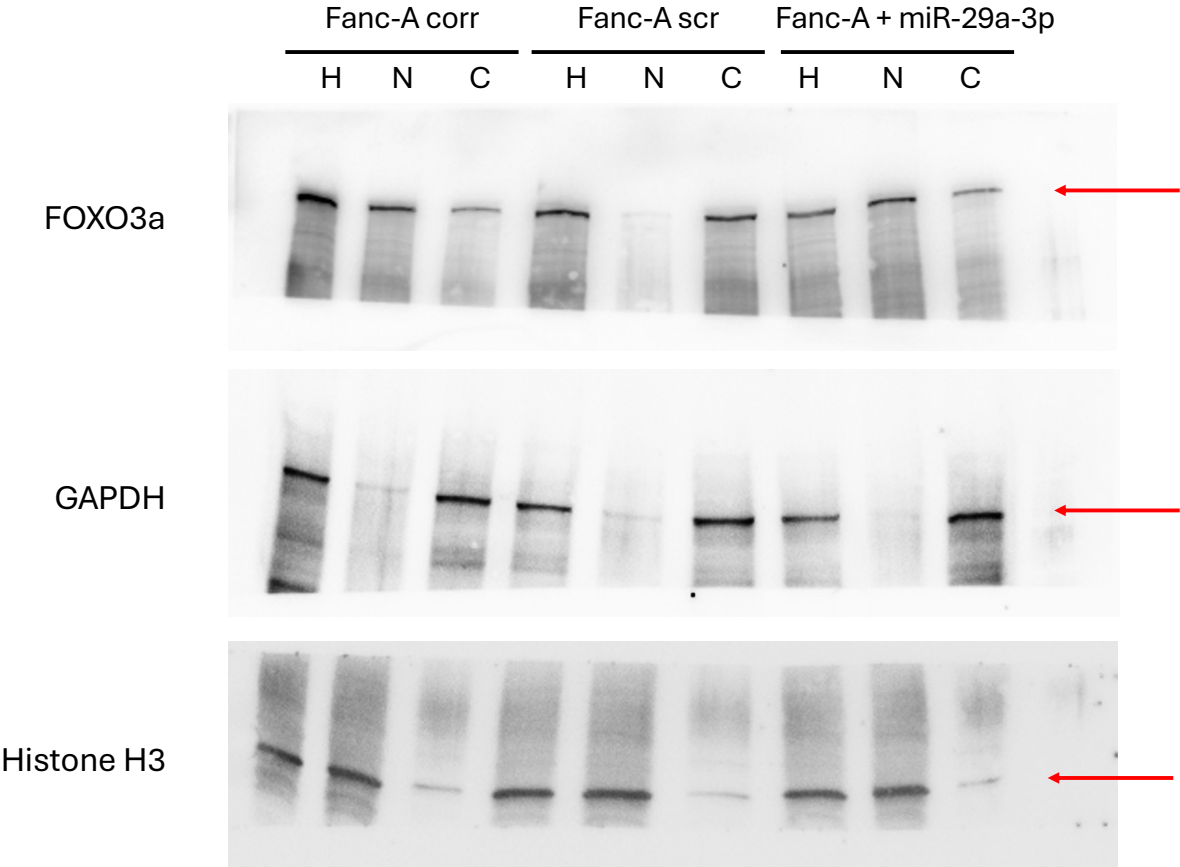

Figure 4

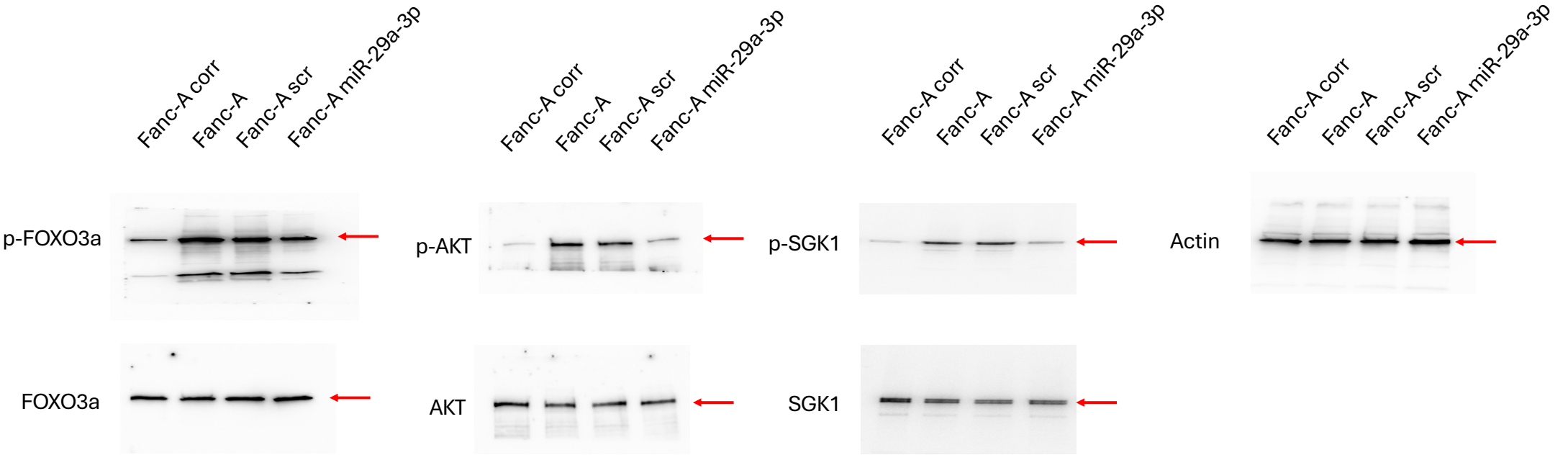

Figure 5

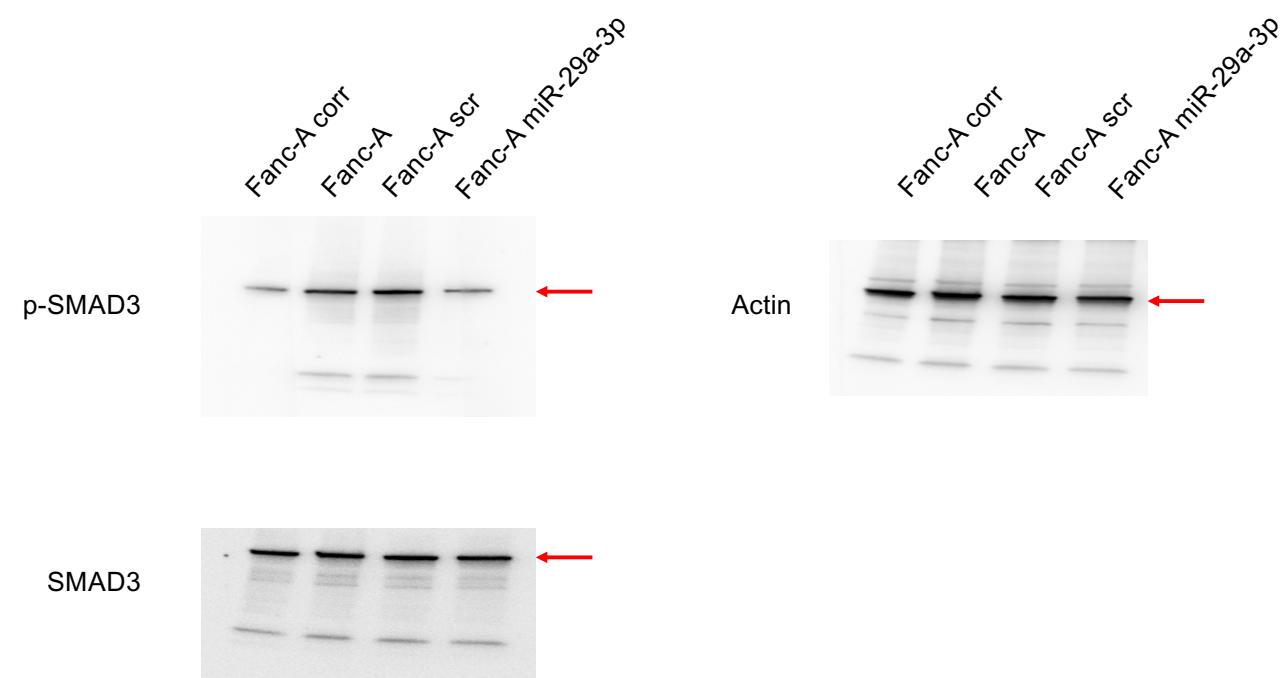

Figure 7

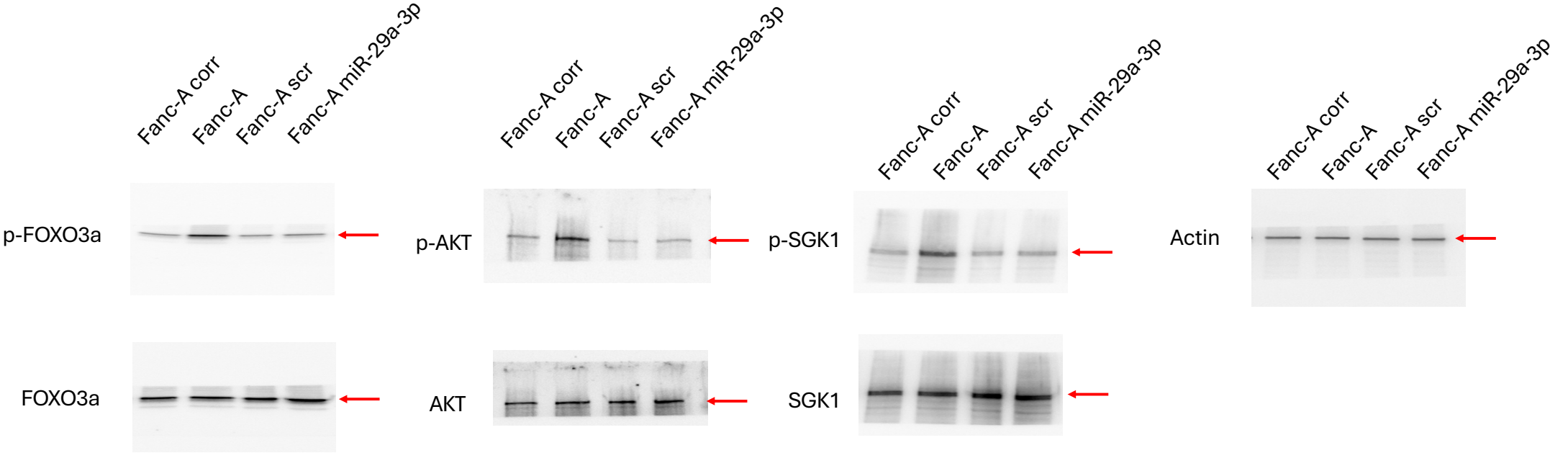

Supplement: Supplementary file 2 — (PDF 1.78 MB) [file 18_2025_5775_MOESM2_ESM.pdf]
